# The Evolutionary History of Ephs and Ephrins: Toward Multicellular Organisms

**DOI:** 10.1093/molbev/msz222

**Published:** 2019-10-07

**Authors:** Aida Arcas, David G Wilkinson, M Ángela Nieto

**Affiliations:** 1 Instituto de Neurociencias (CSIC-UMH), Avda, San Juan de Alicante, Spain; 2 Neural Development Laboratory, The Francis Crick Institute, London, United Kingdom

**Keywords:** Eph receptor, phylogeny, cell–cell contact, cell segregation, RTK pathways, multicellularity

## Abstract

Eph receptor (Eph) and ephrin signaling regulate fundamental developmental processes through both forward and reverse signaling triggered upon cell–cell contact. In vertebrates, they are both classified into classes A and B, and some representatives have been identified in many metazoan groups, where their expression and functions have been well studied. We have extended previous phylogenetic analyses and examined the presence of Eph and ephrins in the tree of life to determine their origin and evolution. We have found that 1) premetazoan choanoflagellates may already have rudimental Eph/ephrin signaling as they have an Eph-/ephrin-like pair and homologs of downstream-signaling genes; 2) both forward- and reverse-downstream signaling might already occur in Porifera since sponges have most genes involved in these types of signaling; 3) the nonvertebrate metazoan Eph is a type-B receptor that can bind ephrins regardless of their membrane-anchoring structure, glycosylphosphatidylinositol, or transmembrane; 4) Eph/ephrin cross-class binding is specific to Gnathostomata; and 5) kinase-dead Eph receptors can be traced back to Gnathostomata. We conclude that Eph/ephrin signaling is of older origin than previously believed. We also examined the presence of protein domains associated with functional characteristics and the appearance and conservation of downstream-signaling pathways to understand the original and derived functions of Ephs and ephrins. We find that the evolutionary history of these gene families points to an ancestral function in cell–cell interactions that could contribute to the emergence of multicellularity and, in particular, to the required segregation of cell populations.

## Introduction

Eph receptors (Ephs) and their ephrin ligands are cell surface bound signaling molecules that have major roles in the establishment and maintenance of tissue organization in vertebrates. These roles are mediated by the regulation of cell adhesion and the actin cytoskeleton that drives the segregation of cells to form sharp borders (Canty et al. 2017; Friedl and Mayor 2017; Taylor et al. 2017), guides neuronal growth cones (Xu and Henkemeyer 2012; Herrera et al. 2019) and migrating cells (Poliakov et al. 2004; Niethamer and Bush 2019), and is required for angiogenesis (Kuijper et al. 2007) and the asymmetric positioning of organs (Cayuso et al. 2016). Disruption of Eph/ephrin signaling contributes to the metastasis of cells during cancer (Pasquale 2010; Bhatia et al. 2019). In addition to having crucial roles in morphogenesis, Eph and ephrin signaling regulate cell differentiation and the maintenance of stem cells in some contexts (Wilkinson 2014). Important insights into how Ephs and ephrins regulate cell behavior have come from studies of their structure and biochemical mechanisms of signaling.

**Fig. 1.**
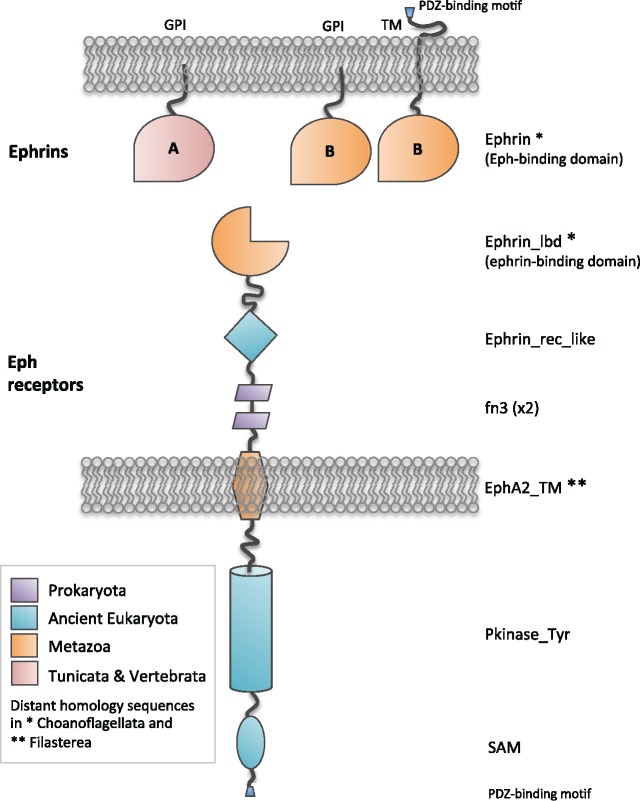
Ephs and ephrins domain architecture and domain emergence in evolution. The Eph-binding domain (Ephrin domain, PF00812) is specific of ephrins and emerged in the metazoan ancestor, being class-B the ancient form while class-A likely emerged in the tunicate ancestor. Class-A ephrins are anchored to the membrane through a GPI linkage, while class-B ephrins have a TM domain and a short cytoplasmic region that is phosphorylated upon receptor binding in vertebrates and tunicates. In nonvertebrates, except for tunicates and some sponge species, class-B ephrins can have either a GPI linkage or a TM domain. The architecture of class-A and -B Ephs is identical. The ephrin-binding domain (Ephrin_lbd, PF01404) and the transmembrane (EphA2_TM, PF14575) domains are found in Metazoa, although we have found distant homologous sequences for both domains in Choanoflagellata (asterisk) and Filasterea (double asterisk), as well as for the Ephrin domain. The other domains are present in a variety of proteins and are of older origin: the fn3 (PF00041) originated in Prokaryota, and the Ephrin_rec_like (PF07699), SAM (PF00536 and PF07647) and Pkinase_Tyr (PF07714) domains emerged in ancient eukaryotes.

Ephs are the largest subfamily of receptor tyrosine kinases (RTKs) in vertebrates (Grassot et al. 2006), with most organisms bearing 14 Ephs that belong to class-A (EphA1–EphA8, EphA10) or class-B (EphB1–EphB4, EphB6) receptors, based on sequence similarity and ligand-binding affinity. Ephs have three main regions (fig. 1): 1) an N-terminal extracellular section composed of a globular ligand-binding domain, a cysteine-rich region and two fibronectin type III (fn3) domains; 2) a transmembrane (TM) segment; and 3) a cytoplasmic region that includes the protein tyrosine kinase (Pkinase_Tyr) domain, a Sterile Alpha Motif (SAM), and a PDZ-binding motif. The ligands for Ephs are named ephrins, and are also organized in class-A (ephrin-A1 to A5) and class-B (ephrin-B1 to B3) in vertebrates (fig. 1). Both classes have an extracellular receptor-binding domain, with class-A ephrins anchored to the membrane through a glycosylphosphatidylinositol (GPI) linkage, whereas class-B ephrins possess a TM domain and a short cytoplasmic region. In general, Ephs promiscuously bind ephrins of their same A or B class. In addition, EphA4 can also bind ephrin-B2 and -B3 (Qin et al. 2010), and EphB2 can bind ephrin-A5 in addition to class-B ephrins (Himanen et al. 2004). This cross-class binding occurs due to structural and compensatory stabilizing interactions between these Ephs and ephrins (Himanen et al. 2004; Guo and Lesk 2014).

As both Ephs and ephrins are tethered to the cell surface, their binding requires cell-to-cell contact. Once ephrins bind to Ephs, Eph/ephrin molecules form heterotetramers, oligomerize, and then assemble in large signaling clusters, the size of which correlates with the strength of the signal (Egea et al. 2005). In contrast to other RTKs (whose ligands are mostly soluble), this binding can not only lead to forward signaling through the activation of Ephs but also reverse signaling through the ephrin-expressing cells (Murai and Pasquale 2003; Pasquale 2008). Forward and reverse signaling can control the actin cytoskeleton through Rho GTPases and the function of cell adhesion molecules to regulate cell repulsion and adhesion (Fagotto et al. 2013; Taylor et al. 2017).

Forward signaling is primarily mediated by the activation of the kinase domain of the receptor (Kullander et al. 2001), which results in the autophosphorylation of the Eph juxtamembrane tyrosine residues and phosphorylation of other proteins. Interestingly, the kinase domain of both EphA10 and EphB6 in vertebrates lacks the residues necessary for catalytic activity, suggesting these might not function by phosphorylating cytoplasmic target proteins (Truitt and Freywald 2011), and instead signal through association with other types of Ephs (Freywald et al. 2002; Johnson et al. 2016). Furthermore, alternatively spliced isoforms identified for many Ephs differ from the typical structure (some lacking the kinase domain; Ciossek et al. 1999), and have altered activity and specific functions (Pasquale 2010). Eph SAM domains play a regulatory role via allosteric coupling to the juxtamembrane region (Kwon et al. 2018) and have target-specific binding properties, partly explaining the different cellular responses elicited after binding the same type of ephrin (Wang et al. 2018).

In reverse signaling that occurs upon formation of Eph/ephrin clusters, the intracellular ephrin-B domain is phosphorylated by kinases of the Src family (Palmer et al. 2002). In addition, the PDZ domain interaction motif of ephrins can mediate signaling that regulates cell morphology (Bochenek et al. 2010; Cayuso et al. 2015) and cell migration (Lee et al. 2006), but this may occur independently of interaction with Ephs. As class-A ephrins do not have an intracellular domain, the tyrosine–protein kinase Fyn or other coreceptors are required to transduce the signal (Davy et al. 1999).

Adding to the complexity of Eph/ephrin mechanisms of action, certain ephrins can induce signaling cascades independently of Ephs (Chin-Sang et al. 2002) and similarly, Ephs can signal independently of ephrins, through the binding to other ligands (Tsuda et al. 2008) or through cross-talk with other receptors, cell surface proteins, and cytoplasmic signaling molecules (Murai and Pasquale 2003). Interestingly, this ephrin-independent signaling can have antagonistic effects to ephrin-dependent signaling (Miao et al. 2009).

Ephs and ephrins have been identified in many metazoan groups and their tissue distribution has been well studied. However, the high complexity of Eph/ephrin interactions, including their promiscuity, makes it very difficult to describe signal transduction pathways downstream of specific family members and ligands. Furthermore, although Ephs and ephrins have important functions in development, it is not clear which of these is ancestral. One possibility is that the prominent role of Eph/ephrin signaling in maintaining tissue organization seen in vertebrates is ancestral. The A and B classes of Ephs and ephrins can regulate distinct cell responses and signal through different pathways (Pasquale 2010; Kania and Klein 2016; Niethamer and Bush 2019), raising the question of how these evolved. Analyzing the evolutionary history could therefore help understand the functions of Ephs and ephrins. Previous evolutionary studies revealed important features (Drescher 2002; Mellott and Burke 2008; Krishnan et al. 2019), but there are many aspects that deserve further investigation. Taking advantage of the wealth of publicly available genomes, we have been able now to include representatives from phyla not used in previous studies, and analyze the presence of Ephs and ephrins and their domains in the whole tree of life. We have also examined the presence of protein domains that correlate with the emergence of functional characteristics specific of particular ligands and receptors, the binding of alternative ligands, and the putative conservation of signaling pathways in representative phyla to understand ancestral and co-opted functions of Ephs and ephrins.

## Results

### Ancestral Ephs and Ephrins

The most ancient bona fide orthologs of Ephs and ephrins have been recently found in Porifera, although they may have an older origin, as homologous sequences have been identified in choanoflagellates (Krishnan et al. 2019), unicellular organisms closely related to metazoans that possess numerous families of RTKs (Manning et al. 2008; Suga et al. 2012, 2014). Nevertheless, an active Eph/ephrin pair has not been detected in this holozoan lineage yet.

**Fig. 2.**
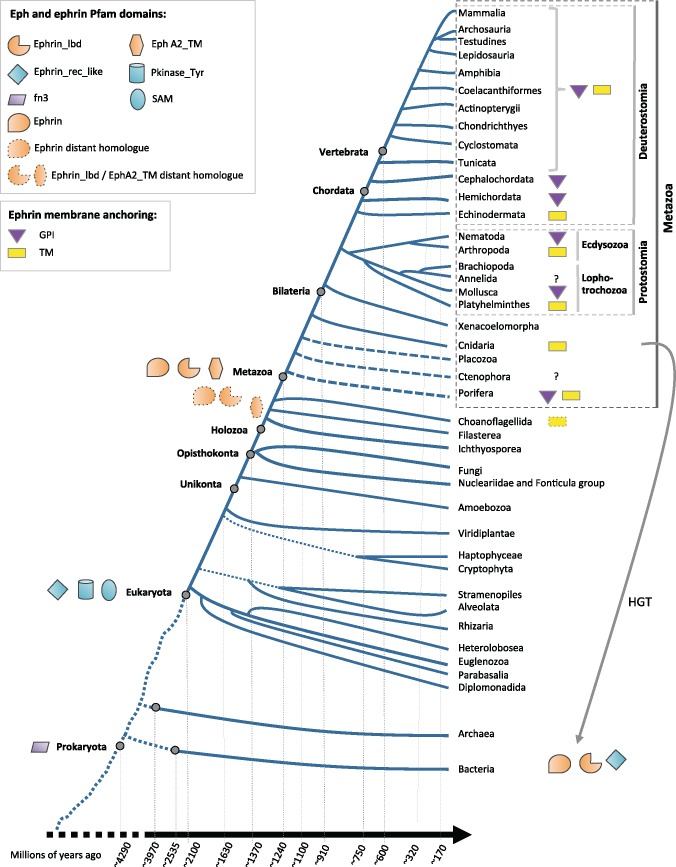
Tree of life showing the phylogenetic groups used in this study. The figure also shows the emergence of Eph and ephrin domains in evolution, the presence in unicellular holozoans of homologs of domains typical of Eph and ephrins, the distribution of GPI and TM structures in ephrins, and HGT events of Eph/ephrins from Metazoa to prokaryotes. Dashed lines to Porifera, Ctenophora and Placozoa indicate the controversy about the position of these phyla relative to Cnidaria and Bilateria. Question marks indicate that the predictors we used for TM and GPI were unable to confidently detect these structures in the ephrin orthologs. Interestingly, we have detected domains that are specific for Eph and ephrins in several bacteria. We found the Eph-binding domain (Ephrin) in two proteins in *Endozoicomonas montiporae*, a gammaproteobacterium isolated from the coral *Montipora aequituberculata* (cnidaria) (Yang et al. 2010). Although we did not detect any TM or GPI-anchor region in the bacterial sequences, these proteins have 33% identity to ephrins in *Nematostella vectensis* (Cnidaria) and nearly 30% to sequences from *Hydra vulgaris* (Cnidaria) and *Strongylocentrotus purpuratus* (Echinodermata), suggesting that *Endozoicomonas montiporae* acquired these sequences from the coral by HGT. With respect to Eph domains, we have found the Ephrin_rec_like domain in the uncharacterized protein YM304_37510 from *Ilumatobacter coccineum* YM16-304, an actinobacterium isolated from seashore sand (Matsumoto et al. 2013). This protein also contains a HYR domain, which belongs to same Pfam clan as the fn3 domain, suggesting that it might be a protein fragment with a domain architecture and structure resembling that of Eph/RTK superfamily members. As such, Phyre2 (Kelley et al. 2015) predicts its 3D structure to be highly similar to that of the human Receptor tyrosine–protein kinase erbB-4, which contains a TM region and a Pkinase_Tyr domain (40% of the bacterial sequence was modeled with 98.7% confidence to sp|Q15303|ERBB4_HUMAN). In addition, the Ephrin_lbd domain, Eph-specific, is present in an uncharacterized protein (ADIWIN_0470) in the flavobacterium *Winogradskyella psychrotolerans* RS-3, isolated from Arctic sediment (Begum et al. 2013). All of the above also seem to be result of HGT (gray arrow).

**Fig. 3.**
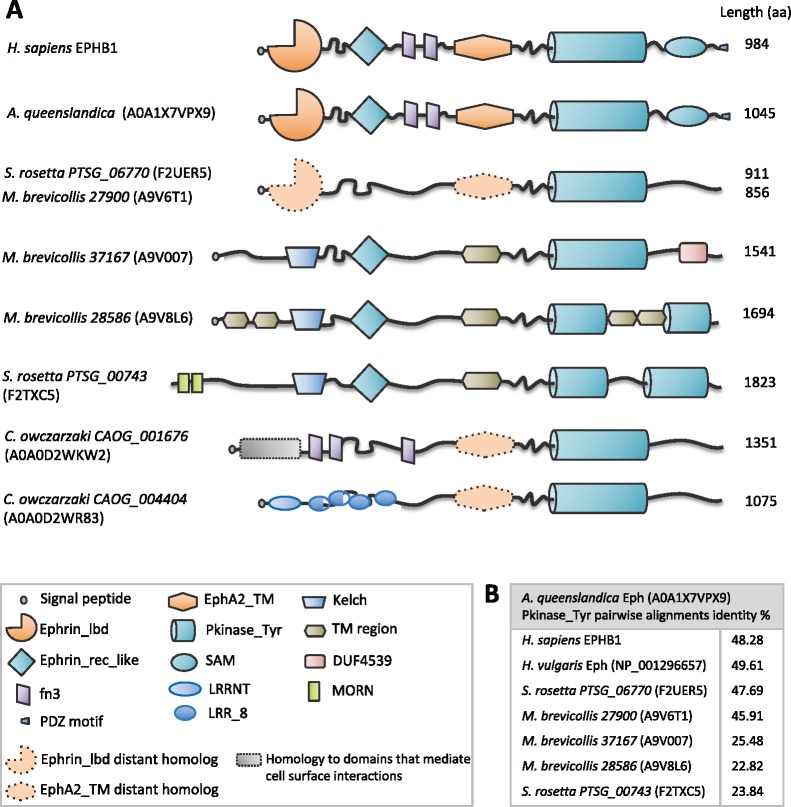
Putative Eph homologs in choanoflagellates. (*A*) The overall domain architecture of Ephs has not changed within Metazoa. We have found several sequences in Choanoflagellata and Filasterea that share part of the domain architecture with metazoan Ephs, such as the protein of unknown function A9V007 (gene *37167*) and the predicted protein A9V8L6 (gene *28586*) both in *Monosiga brevicollis* or the TKL protein kinase (F2TXC5, gene *PTSG_00743*) in *Salpingoeca rosetta.* We have also identified Eph-like sequences in *S. rosetta PTSG_06770* (F2UER5) and *Monosiga brevicollis 27900* (A9V6T1) with distant homology to the ephrin-binding (Ephrin_lbd) and EphA2_TM domains. The filasterean *Capsaspora owczarzaki* genome already encodes proteins with the core Eph domain structure, and homology in N-terminal to domains involved in cell–cell interaction. This schematic representation does not show the actual proportion of the proteins or the domains. LRRNT, Leucine-rich repeat N-terminal domain; LRR_8, Leucine-rich repeat; Kelch, Kelch motif; DUF4539, Domain of unknown function DUF4539; MORN, Membrane Occupation and Recognition Nexus. (*B*) Pairwise alignment identity % between *Amphimedon queenslandica* Eph Pkinase_Tyr domain and the Pkinase_Tyr domain from sequences in other species. The percentage of identity is similar between the sponge and human Eph, and between the sponge and the Eph-like sequences in Choanoflagellata.

To extend previous phylogenetic analyses, we used Blast and Hidden Markov Model algorithms to search the genomes of holozoans, where many families of tyrosine kinases have been described (Suga et al. 2014). We have found sequences with distant homology to Ephs in choanoflagellates and Filasterea (figs. 1–3), confirming the findings by Krishnan et al. (2019). The sequences encoding the closest Pkinase_Tyr domain to that in Ephs are found in *Salpingoeca rosetta* F2UER5 (gene *PTSG_06770*) (Fairclough et al. 2013) and *Monosiga brevicollis* A9V6T1 (gene *27900*) (fig. 3*A*). Both have nearly 50% sequence identity to the Pkinase_Tyr domain in Porifera (*Amphimedon queenslandica*) Eph (fig. 3*B*). Using HHPred (Alva et al. 2016), we have also detected regions with distant homology to the Ephrin_lbd and Epha2_TM domains (probability = 72.2% and 90.5%, *P* value = 1.1e-3 and 9.4e-6, respectively, for F2UER5 and A9V6T1). Although the lack of synteny between choanoflagellate and metazoan genomes makes establishing direct orthology difficult, these proteins suggest that the common metazoan–choanoflagellate ancestor had an ancestral Eph.

As the Eph-binding domain (termed Ephrin domain in Pfam, PF00812) in ephrins resembles the fold of cupredoxin proteins (Himanen et al. 2001), using HHPred, we searched for proteins with this fold in choanoflagellates and Filasterea. Interestingly, we detected the uncharacterized protein F2TW21 (gene *PTSG_11572*) in *S. rosetta*, which contains a region with distant homology to the Eph-binding domain (probability = 81.6%, *P* value = 2.1e-4) and a TM domain similar to Glycophorin_A (PF01102) (probability = 80.1%, *P* value = 3.6e-5). To discard that this protein is a cupredoxin, we used IonCom (Hu et al. 2016), a predictor of protein-binding sites for ions, and it did not find any for copper, as the ephrin-like sequence lacks the residues needed for copper binding but has the cysteines required for cross-sheet disulfide bridge formation that are found in metazoan ephrins. We also did 3D models of choanoflagellate cupredoxins with Phyre2 (Kelley et al. 2015), for example, *S. rosetta* F2URU3 (gene PTSG_10601), which has a Cu_bind_like domain (PF02298) and its fold is completely different (see supplementary fig. S1, Supplementary Material online) from that predicted for the ephrin-like sequence we have found. The equivalent residues in the cupredoxin (supplementary fig. S1*G*, Supplementary Material online) are not exposed.

Given that *S. rosetta* has a pair of Eph- and ephrin-like sequences, we wondered whether these proteins might interact. Our 3D models of the two proteins compared with structures from human Ephs and ephrins (supplementary fig. S1*A, B, D, and E*, Supplementary Material online) obtained from the Protein Data Bank (PDB) database (Burley et al. 2017) showed that similarly to metazoan Ephs, the Eph-like protein in *S. rosetta* forms a pocket region where the ephrin-like might bind (supplementary fig. S1*C*, Supplementary Material online). Similarly, like in metazoans, the choanoflagellate ephrin protein has a protruding loop (supplementary fig. S1*F*, Supplementary Material online) that could interact with the Eph-like pocket. These structural analyses show that a binding reaction between the Eph- and ephrin-like sequences from *S. rosetta* may occur.

In summary, this is the first finding of an Eph-like and an ephrin-like pair in the same choanoflagellate species, and structural comparisons show that an interaction between these two proteins might be possible, suggesting that an Eph/ephrin-like binding interaction may already occur in Choanoflagellata.

### Domain Composition of Eph and Ephrin Proteins

To better understand the emergence and possible co-opted functions of Ephs and ephrins, we analyzed the origin and distribution of their different protein domains (as defined by Pfam) (figs. 1 and 2). We have identified the Eph receptor-binding domain only in ephrins. With respect to their membrane domains, we searched for the GPI-anchor and TM regions in other proteins (see Materials and Methods) and did not find relevant hits, but these sequences may be too short to provide relevant information.

Regarding Ephs, both their ligand-binding domain (Ephrin_lbd) and the Eph TM domain (termed EphA2_TM in Pfam, although it is generic to all family members; see Bocharov et al. 2010), seem to be specific to Ephs in vertebrates. As mentioned earlier, sequences related to both are already present in choanoflagellates, and we have also detected sequences with distant homology to the EphA2_TM domain in Filasterea (fig. 2). The other domains in Ephs are more widely distributed, for example, the fn3 is found in the three domains of life and was probably present in the last universal common ancestor. This domain is involved in cell surface binding and is located in a wide variety of extracellular proteins and receptors. The putative ephrin-receptor like domain (Ephrin_rec_like) already emerged in ancient eukaryotes, and although its function is unknown, it is also present in complement and adhesion proteins (Tu et al. 2006; Sato-Nishiuchi et al. 2012). Confirming previous studies (Kim and Bowie 2003; Arcas et al. 2013), the SAM and the Pkinase_Tyr domains are present in a wide variety of eukaryotic proteins and in few bacterial groups, suggesting that prokaryotes might have acquired the SAM by horizontal gene transfer (HGT), as already suggested for the protein tyrosine kinase domain (Arcas et al. 2013). Interestingly, we have also found putative HGT events of Eph- and ephrin-specific domains to prokaryotes (fig. 2). The question remains as to which functions these Eph/ephrin-like proteins are performing in prokaryotes and if they confer some advantage to the bacteria that have retained them.

In brief, the overall structure of human Ephs was already established in the metazoan ancestor, where premetazoan Eph-like sequences and domains of ancient origin were combined with novel domains (fig. 1) allowing them to perform new and specific functions. This likely allowed the interaction of Ephs with new proteins, the ability to elicit new responses, the fine-tuning of particular processes, and the cross-talk of different signaling pathways.

### Origin of Eph-like and Ephrin-like Sequences

As most domains in metazoan Ephs have an ancestral origin, we examined when the specific arrangement of domains found in metazoans occurred. We used the sequences from *S. rosetta* and *Monosiga brevicollis* to search for homologs in other unicellular holozoans through reciprocal Blasts and HMM profiles of the different protein domains. In agreement with recent data by Krishnan et al. (2019), whose work was independent of ours, we did not find any ephrin-like or a complete Eph-like receptor in lineages more ancient than Choanoflagellata.

Nevertheless, we found that Filasterea is the most ancient lineage to have the central domain architecture typical of Ephs (fig. 3*A*), and although these sequences lack the ephrin-binding domain, in their N-terminal region they have homology to domains that mediate cell surface interactions. For example, using HHPred, we have detected homology to the N-terminal region of contactins in *Capsaspora owczarzaki* tyrosine kinase-like (TKL) protein kinase A0A0D2WKW2 (gene CAOG_001676), known to mediate cell surface interactions during nervous system development. Another TKL protein kinase from *Capsaspora owczarzaki*, A0A0D2WR83 (gene CAOG_004404), has several N-terminal leucine-rich repeats, which are involved in protein–protein interaction and are often flanked by cysteine-rich domains (Ephs have a cysteine-rich region between the ligand-binding domain and the fn3 repeats). Thus, as ephrins emerged later in evolution (probably from a cupredoxin that lost the ability to bind copper in the choanoflagellate ancestor; Krishnan et al. 2019), the proto-Eph-like sequences present in Filasterea may bind other ligands, and also participate in cell–cell adhesion, albeit using different signaling pathways.

In summary, the proto-Eph-like sequences in Filasterea, although lacking the ephrin-binding domain, may be active receptors able to bind other types of ligands. These sequences may be ancestral precursors of the Eph-like sequences in Choanoflagellata or divergent homologs of a common holozoan ancestor.

### Evolutionary History of Ephs and Ephrins

**Fig. 4.**
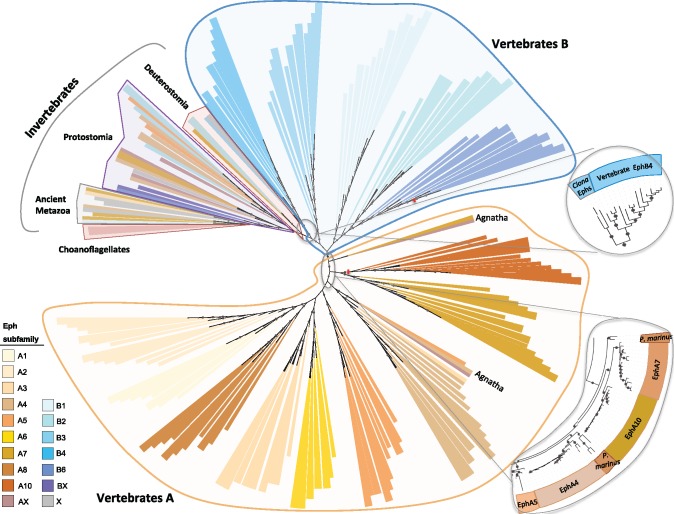
Phylogenetic tree of the ligand-binding domain (Ephrin_lbd) in Ephs reconstructed by Bayesian inference. In this schematic view, the tree is unrooted and branch length is not considered for an easier visualization. For a detailed view of the tree identifying each branch, see supplementary figure S5, Supplementary Material online. Model of amino acid evolution: JTT+I + G. About 251 sequences from 41 species are included in the tree. Sequences are annotated as predicted by InParanoid and have been colored accordingly. We have included into subfamily X those sequences whose best hits were to both class-A and -B human Ephs, and into subfamily AX or BX those sequences with equal distance to homologs from various subfamilies within class-A or class-B Ephs, respectively. Red lines in EphA10 and EphB6 branches mark the loss of kinase activity in these receptors. Choanoflagellate sequences are close to invertebrate metazoan species, which group similarly to the taxonomic tree: ancient Metazoa, Protostomia and Deuterostomia, with sequences from the latter near the branching point of vertebrate Ephs. Two Eph sequences from the ascidian *Ciona intestinalis* group with vertebrate EphB4 sequences (magnification on the right), pointing to a subfunctionalization of some Tunicata receptors to bind only class-B ephrins. The magnification on the bottom right shows there are two groups of agnathan orthologs of vertebrate class-A Ephs, suggesting that part of the expansion of EphAs observed in vertebrates occurred after the split of Agnatha.

**Fig. 5.**
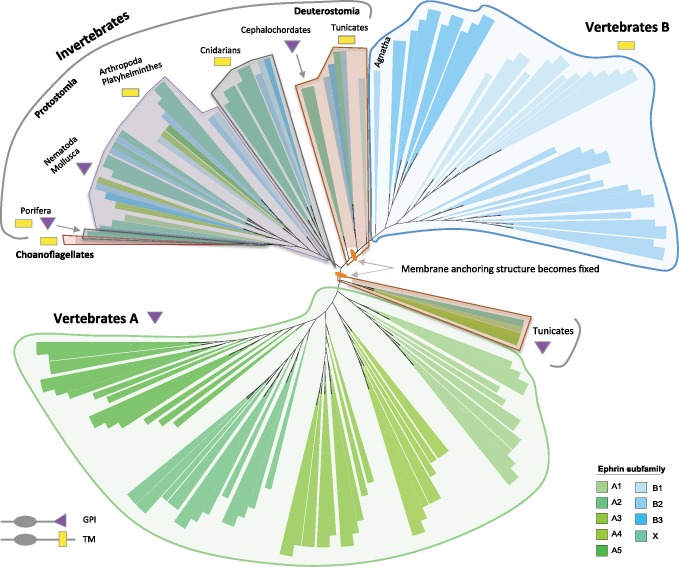
Phylogenetic tree of ephrins reconstructed by Bayesian inference. In this schematic view the tree is unrooted, and branch length is not considered for an easier visualization. For a detailed view of the tree identifying each branch, see supplementary figure S8, Supplementary Material online. Model of amino acid evolution: JTT+I + G. About 164 sequences from 41 species are included in the tree. Sequences are annotated as predicted by InParanoid and have been colored accordingly. We have included into subfamily X those sequences, whose best hits were to both class-A and -B human ephrins. Choanoflagellate sequences are close to Porifera and other ancient metazoan species, and invertebrate deuterostomians group close to vertebrate sequences. Class-A and -B ephrins of vertebrates and tunicates appear clearly differentiated in opposing sides of the tree. The orange dots mark the point in evolution when GPI and TM were class-fixed. In invertebrate ephrins, there is tinkering of GPI and TM structures in the different phyla (see fig. 2). Although the overall sequences from cephalochordates and hemichordates are closer to those in vertebrate class-B ephrins, they contain a GPI structure.

Previous phylogenetic studies (Drescher 2002; Mellott and Burke 2008; Brunet et al. 2016) using the available data sets already provided significant information about the evolution of Ephs and ephrins. At present, it is possible to search for orthologs of Ephs and ephrins in species representative of all main phylogenetic groups (see supplementary table S1, Supplementary Material online). Thus, we have built phylogenetic trees using Bayesian Inference (BI) and Maximum Likelihood method (ML) (see figs. 4 and 5, and supplementary figs. S2–S9, Supplementary Material online), both with or without the Eph- and ephrin-like sequences from choanoflagellates as outgroup. Both methods generated trees with similar topologies, and have allowed us to reveal new members, confirm some of the previous proposals and to reach robust evolutionary conclusions.

In the case of Ephs, we built phylogenetic trees with the whole Eph protein or only with the ephrin-binding domain (Ephrin_lbd; fig. 1), because we were interested in the evolution of the interaction with ephrins. Both gave very similar results (supplementary figs. S2–S5, Supplementary Material online). Eph sequences from invertebrates group at the base of the tree (fig. 4 and supplementary figs. S2–S5, Supplementary Material online) and phyla are clustered similarly as in the tree of life (ancient Metazoa, Protostomia, and Deuterostomia), with hemichordates and chordates near the branching point of vertebrate class-B Ephs. This indicates that the ancestral Eph receptor belonged to this class. Indeed, two Eph sequences from the ascidian *Ciona intestinalis* group with EphB4 sequences from vertebrates (highlighted on the right hand side of fig. 4, and supplementary figs. S3–S5, Supplementary Material online), which suggests that some Eph receptors in Tunicata had already specialized into binding class-B ephrins only, and that *ephB4* may represent the most ancient vertebrate Eph. Consequently, *ephB4* could be the Eph of choice for comparative functional studies with invertebrate model organisms such as *Drosophila*.

In agreement with a previous study (Mellott and Burke 2008), we confirm that the most ancient vertebrate class-A receptors are *ephA10*, a kinase-dead receptor (Truitt and Freywald 2011), and *ephA7* (fig. 4 and supplementary figs. S2–S5, Supplementary Material online). On the other hand, the most modern is *ephA1*, which emerged by gene duplication at the base of sarcopterygian species (a group that includes the coelacanth and tetrapods) and thus absent from Chondrichthyes and actinopterygian fish. Another vertebrate Eph phylum-specific duplication occurred in Amphibia, as *Xenopus tropicalis* contains duplicates of *ephA4* and *ephB1*. In addition, several gene losses have happened along the vertebrate evolutionary tree. For example, after the teleost additional whole genome duplication, several Eph copies were lost in some sublineages, and similarly, *ephB4* and *ephA10* have been lost in turtles and in Lepidosauria, respectively.

In the lamprey *Petromyzon marinus*, we have found only five class-A and two class-B Eph sequences (probably corresponding to EphA4, A5, A7, A8, A10, B2, and B6), which group at the base of other vertebrate Ephs in our phylogenetic tree (highlighted at the bottom right of fig. 4 and also see supplementary figs. S2–S5, Supplementary Material online), suggesting that part of the expansion of EphAs observed in vertebrates (Brunet et al. 2016) might have occurred after the split of Agnatha. However, we cannot exclude that the absence of other orthologs may be due to the incomplete genomes currently available for these jawless vertebrates.

With respect to ephrins, the phylogeny (fig. 5 and supplementary figs. S6–S9, Supplementary Material online) suggests that ancient ephrins have a B-type ligand-binding domain, which is supported by the fact that the original Eph receptor seems to be class-B also (fig. 4 and supplementary figs. S2–S5, Supplementary Material online), but importantly, they have this ligand-binding domain regardless of whether they have TM or GPI structures. For instance, the ephrin homologs in sponges have a GPI-anchor structure, a TM region or both in the same sequence (Krishnan et al. 2019) but cnidarian ephrins contain a TM region conserved in all paralogs even after their huge expansion in this phylum. Within Protostomia, the ephrin in molluscs and nematodes has a GPI-anchor while in platyhelminthes and arthropods it has a TM region. In Deuterotostomia, echinoderms also have a TM region in their ephrin, whereas hemichordates and cephalochordates have a GPI structure (see fig. 2). Tunicata is the oldest phylogenetic group where we identified class-A ephrins, and they are always associated with GPI-anchors. Similarly, class-B ephrins are only associated with TM structures (fig. 5 and supplementary figs. S6–S9, Supplementary Material online), as previously observed (Mellott and Burke 2008). Intriguingly, while in the ascidian *C. intestinalis*, there is one ephrin with a TM region and a cluster of four ephrins with a GPI-anchor (that was generated through a local duplication specific of this genus), in *Oikopleura dioica* (Appendicularia) it is the other way around. These differences, also observed in other gene families such as caspases and notochord genes (Weill et al. 2005; Kugler et al. 2011), might be related to the very different developmental patterns and life cycle of these two tunicate groups. Overall, these results indicate there has been shuffling of GPI and TM structures in ephrins in nonvertebrate metazoans up to tunicates, where these anchoring structures are class-fixed, as they are in the vertebrate ephrins. Therefore, the C-terminal region of ephrins does not define their class and thus, only the ligand-binding domain should be used to this purpose.

Leaving apart the Eph-binding domain (fig. 1), the sequence and structure of ephrins vary in metazoan phyla. As mentioned earlier, Porifera may have ephrins with GPI-anchor and a TM region in the same sequence (Krishnan et al. 2019). In Arthropoda, the intracellular region is very short compared with that in other phylogenetic groups, and in *Drosophila melanogaster* and *Hymenolepis microstoma*, a platyhelminth, their ephrin sequences contain three and two TM domains, respectively. Interestingly, even though the cytoplasmic C-terminal region in *Hymenolepis microstoma* ephrin is very long, our profile of class-B ephrin sequences (see Protein Domain Identification in Materials and Methods) still returned this ephrin as a hit, showing its similarity to class-B ephrins and suggesting that reverse signaling is already present in platyhelminths.

Similar to the situation with the Ephs, the enormous expansion of ephrin representatives in vertebrates seems to have happened after the divergence of Agnatha (fig. 5 and supplementary figs. S6–S9, Supplementary Material online), as we have found only two ephrins in the lamprey *P. marinus* (probably ephrin-B2 and -B3). *efnB3* seems to be missing in Archosauria and *efnA4* in some Lepidosauria. In Gnathostomatha, *efnA1*, *efnA3*, and *efnA4* are part of a genomic cluster, while the other ephrins (and Ephs) are located in different chromosomes or in remote regions within the same chromosome. This suggests that there is no selective pressure to maintain Eph/ephrin genes in genomic clusters.

In summary, Ephs and ephrins diversified at different points in evolution as already suggested (Mellott and Burke 2008). Ephrins had already differentiated in the common ancestor of tunicates and vertebrates, but class-A Ephs did not emerge before the vertebrate lineage. Importantly, ancient Ephs were likely class-B receptors, and ancient ephrins have a B-type ligand-binding domain, but that is independent of containing a GPI-anchor or TM. Class-A ephrins binding to the membrane with GPI and class-B ephrins having a TM domain was fixed only from tunicates, suggesting that class-specific Eph/ephrin-binding predates vertebrates. As EphAs are vertebrate-specific, both class-A and -B ephrins likely bind to EphB receptors in tunicates, although some *C. intestinalis* receptors that in the phylogenetic tree group with vertebrate EphB4 (highlighted on the right of fig. 4, and supplementary figs. S3–S5, Supplementary Material online) might bind only class-B ephrins. Coevolution analysis with MirrorTree (Ochoa and Pazos 2010) using a reduced set of sequences representing all taxa (see Materials and Methods) showed that Eph receptors and ephrin ligands tend to coevolve (data not shown), although there are differences among taxa and certain vertebrate sequences, probably due to their cross-class-binding ability and the fact that several Ephs also bind ligands other than ephrins (see below cross-class-binding section). Different lineage-specific expansions (cnidarians, nematodes, tunicates, and vertebrates) and losses have shaped the Eph/ephrin repertoire in metazoans. The huge expansion in vertebrates correlates with an increased diversity of cell types and the enlargement of intracellular signaling pathways and in particular, cell–cell interactions.

### Alternative Ligands for an Orphan Receptor

We have found *Eph* genes in the Placozoa genomes (*Trichoplax adhaerens* and *Hoilungia hongkongensis*; Eitel et al. 2018) but no ephrins. Thus, as the Eph/ephrin pair was already present in the metazoan ancestor, placozoans secondarily lost the ephrin and their Ephs should bind to at least one alternative ligand. When we searched for described alternative ligands for Ephs, we found one ortholog of the gene encoding vesicle-associated membrane protein-associated protein (VAP), shown to bind Ephs in *Drosophila melanogaster* (Pennetta et al. 2002), *Caenorhabditis elegans* (Cheng et al. 2008), and human (Tsuda et al. 2008) and to regulate lipid transport, the unfolded protein response, membrane trafficking (Lev et al. 2008), the stabilization of microtubules (Wang et al. 2014) and leaf senescence in *Arabidopsis* (Ichikawa et al. 2015). VAP proteins contain among other domains an N-terminal major sperm protein (MSP) domain (Lua et al. 2011), which is cleaved from the TM domain and secreted into the extracellular environment, where it binds to different receptors and competes with ephrins for Eph binding (Tsuda et al. 2008). The identified VAP ortholog in *Trichoplax* conserves the whole domain architecture and the residues in the MSP domain described to bind to EphA4 in human (Lua et al. 2011), indicating that *Trichoplax* may use VAP as a ligand for Ephs.

### Kinase-Dead Ephs Can Be Traced Back to Gnathostomata

There are three critical motifs necessary for catalytic activity of the Eph kinase domain: the VAIK motif in subdomain II, the HRD at the phosphotransfer site in subdomain V, and the DFG motif in subdomain VII, required for coordinating the β and γ phosphates of ATP. We analyzed the conservation of these motifs in the different Eph orthologs (see supplementary fig. S10, Supplementary Material online), and found that from Porifera (*Amphimedon queenslandica*) to human, the three motifs are present in all phyla, indicating that Ephs were kinase-active since their emergence. However, alterations in both EphA10 and EphB6, render both receptors kinase-dead (Matsuoka et al. 1997; Truitt and Freywald 2011). Orthologs are found down to Gnathostomata, and while all EphA10 are kinase-dead, EphB6 is kinase-inactive only in mammals. In summary, human kinase-dead Ephs can be traced back to the ancestor of Gnathostomata, although EphB6 is kinase-inactive only in mammals. Whether an *ephA10* ortholog is also present in Agnatha will hopefully be determined when new and more complete assemblies of *P. marinus* or other lamprey species become available. Regarding invertebrates, some phylogenetic groups have Ephs with isoforms whose kinase activity is altered, indicating that the existence of Ephs with altered kinase activity together with those that can fulfill canonical signaling may have some beneficial effect, such as fine-tuning activity levels.

### Eph/Ephrin Cross-Class Binding Is Specific to Gnathostomata

EphA4 and EphB2 can fulfill cross-class binding (Himanen et al. 2004; Qin et al. 2010), and previous studies have analyzed the molecular basis for this. For instance, EphA4 can bind class-A ephrins plus ephrin-B2 and -B3 due to the conformational plasticity of the receptor in its ligand binding side, which recapitulates structural hallmarks of class-B Ephs upon binding ephrin-Bs (Bowden et al. 2009). However, EphA4 cannot bind ephrin-B1 due to a change in the amino acids located in the interface region (Guo and Lesk 2014). We have analyzed the sequences in all vertebrates, and found that the main residues involved in the binding of ephrin-B2 and -B3 to EphA4 are only conserved in Gnathostomata (see supplementary fig. S11A, Supplementary Material online).

In the lamprey, the sequence to bind ephrin-Bs is not entirely conserved in EphA4 (supplementary fig. S11*B*, Supplementary Material online), and besides, its ephrin-B3 does not have the Leu and Trp residues needed for the binding to the receptor, and its ephrin-B2 ortholog lacks the region of interaction. This suggests that cross-ligand binding mediated by EphA4 does not occur in lampreys. Interestingly, ephrin-B2 reverse signaling after EphA4 binding is important during the formation of intersomitic boundaries in the chick embryo and very likely in all vertebrates (Durbin et al. 1998; Rawls et al. 2000; Watanabe et al. 2009; Hester et al. 2011), and somite morphology and development is different in the lamprey (Hammond et al. 2009).

To get further insight into how the Eph/ephrin cross-class binding was achieved in Gnathostomata, we used CoeViz (Baker and Porollo 2016), to inspect putative coevolution of protein residues considering intraprotein residue–residue contacts. We examined the conservation and changes of pairs and clusters of residues involved in EphA4–ephrinB2 (and -ephrinB3) binding. Several of the main interacting residues (see supplementary fig. S11*B*, Supplementary Material online) in EphA4 in the agnathan *P. marinus* have biochemical properties that differ from those in Gnathostomata (supplementary fig. S12*A*–*C*, Supplementary Material online), suggesting that several changes were needed for EphA4 to increase the conformational plasticity of its catalytic pocket to be able to bind class-B ephrins. With respect to ephrin-B2, the most important residues involved in the interaction with EphA4, L121, and W122, are not conserved in *P. marinus* (supplementary fig. S11*A*, Supplementary Material online). However, the CoeViz-predicted coevolving residues (supplementary fig. S12*D*, Supplementary Material online) are all conserved (supplementary fig. S11*A*, Supplementary Material online). These results suggest that the conformational structure of ephrin-B2 and -B3 was already established in Agnatha and a few further changes were needed to establish the cross-class interaction.

Regarding the EphB2/ephrin-A5 binding, compensatory stabilizing interactions occur that are not possible with other class-A ephrins (Himanen et al. 2004). The residues involved in this cross-class binding are present in all gnathostomes (supplementary fig. S13*A* and *B*, Supplementary Material online), but since we have not found any clear class-A ephrin in *P. marinus*, it seems that again, cross-class binding does not occur in Agnatha.

In summary, the Gnathostomata ancestor already had the residues required for Eph cross-class binding. In tunicates, the relevant residues are not conserved, and this type of binding does not seem to happen in Agnatha, which EphA4 catalytic pocket does not have yet the required conformational plasticity to bind class-B ephrins. Thus, cross-class binding is specific to Gnathostomata.

### Sponges and Forward and Reverse Signaling

Forward signaling has been considered the ancestral signaling mode because in Porifera only Ephs had been found (Srivastava et al. 2010; Kania and Klein 2016). However, the finding of ephrins in poriferan genomes (Krishnan et al. 2019 and this work) suggests that both forward and reverse signaling can potentially occur in sponges and other early branching metazoan phyla, also supported by the fact that Ephs and ephrins have the motifs required to link them to these pathways. For example, we found orthologs of most of those genes in both *Amphimedon queenslandica* (>70%), and in *Trichoplax adhaerens* (see supplementary fig. S14*A*–*F*, Supplementary Material online). In the latter, there are fewer genes involved in reverse signaling (supplementary fig. S14*D*, Supplementary Material online), concomitant with the lack of ephrins in this organism. In *Mnemiopsis leidyi*, we have detected few orthologs, which could also be explained by secondary losses (Ryan 2014).

### Rudimental Eph/Ephrin Signaling in Choanoflagellates

As we have identified an Eph/ephrin-like pair in the choanoflagellate *S. rosetta*, we also searched for homologs of Eph/ephrin signaling pathways, and found that these organisms already have about half of the genes described in human (supplementary fig. S14*A*–*F*, Supplementary Material online), more than doubling the number of homologs we detected in Filasterea, which have proto-Eph-like receptors without an ephrin-binding domain. Altogether, this suggests that rudimental Eph/ephrin signaling may occur in choanoflagellates. However, although several RTK homologs, such as Src, have already been described in unicellular Holozoa (Suga et al. 2012, 2014), we detected only few homologs involved in Eph/ephrin signaling in the proteomes of holozoans more ancient than choanoflagellates.

## Discussion

In this work, we have studied the origin and evolutionary history of the Eph/ephrin families by analyzing their presence in the whole tree of life, the emergence of protein domains that can account for functional characteristics specific of certain ligands and receptors, and the appearance and conservation of signaling pathways in representative phyla, which helps to understand the Eph and ephrins ancestral and co-opted functions.

### The Ancestral Eph and Ephrins: Toward the Multicellular Organism

We found that rudimental Eph/ephrin signaling may predate the metazoans, as it may already exist in choanoflagellates. Although Filasterea is the most ancient lineage to have the central domain architecture typical of Ephs, instead of the ephrin-binding domain they have sequences homologous to other domains that mediate cell surface interactions. It is only in Choanoflagellata, where we have identified Eph-like sequences with regions similar to the ephrin-binding (Ephrin_lbd) and Epha2_TM domains, plus a characteristic Pkinase_Tyr domain (fig. 3). Furthermore, in *S. rosetta*, we have also found a sequence compatible with an ephrin to act as a ligand (supplementary fig. S1, Supplementary Material online). Moreover, choanoflagellates have homologs of about half of the genes involved in Eph/ephrin signaling pathways in human. Overall, this indicates that, although the function of these proteins is yet to be determined, some type of rudimental Eph/ephrin interaction upon cell–cell contact may be possible in premetazoan holozoans. Cell–cell contact is indeed compatible with the existence of multicellular colonies in choanoflagellates, and the observed upregulation of genes involved in metazoan cell communication including cadherins during the initiation of colony formation (Fairclough et al. 2013). The later emergence and evolution of stable cell–cell adhesion mechanisms allowed for the transition from unicellular organisms to the multicellular metazoan ancestor (Abedin and King 2010). Indeed, cadherins and β-catenin, essential components of adherens junctions, were already present in Porifera (Nichols et al. 2012) and later, in metazoans, cadherins co-opted domains to mediate new cell–cell interactions (Abedin and King 2008) and constitute the cadherin adhesome network with increased complexity (Murray and Zaidel-Bar 2014).

Eph/ephrin interactions were in place in the metazoan ancestor, as sponges contain the first bona fide receptor/ligand pair, and have the majority of downstream molecules associated with their signaling pathways (supplementary fig. S14, Supplementary Material online). It therefore seems that in basal metazoans Ephs can elicit forward signaling with tyrosine kinase activity upon binding to ephrins or to VAP. The latter does not imply cell–cell interaction, but rather a more canonical receptor-soluble ligand interaction typical of the vast majority of RTKs, as VAP is cleaved and secreted to bind Ephs (Tsuda et al. 2008). Reverse signaling may be possible, at least in sponges. However, as Placozoans secondarily lost the ephrin and, in Ctenophora, the complement of signaling molecules is very much reduced, the most likely scenario is that reverse signaling was fixed later on in evolution (see below).

From all of the above and considering the requirement of cell–cell contact for Eph/ephrin signaling, their ancestral function may be associated with cell–cell communication. As such, in nonvertebrate metazoans (with the exception of tunicates), Ephs (all type-B) bind ephrins regardless of whether they bear a GPI-anchoring or a TM domain, compatible with an initial role of these structures in simply attaching the ephrin to the membrane, which is required for Eph activation and to act as a short-range signal. This may work independently of the type of anchoring, explaining the variability in the selection of both types of membrane anchoring found in early evolution. A different issue is how GPI-anchoring or TM domains influence signaling. Class-B ephrins trigger reverse signaling via tyrosine phosphorylation and via interactions with their C-terminal PDZ-binding motif (Kullander and Klein 2002). In all vertebrate ephrins-B, this motif is “YKV” and remarkably, some ephrins with TM regions from Tunicata but also Cnidaria have a V–X–V motif at their C-terminus, which complies with the characteristics of a PDZ-binding motif (Songyang et al. 1997). This suggests that reverse signaling may already occur in these phylogenetic groups, therefore including diploblasts. The evolution of ephrin-B reverse signaling might underlie new roles that caused fixation of ephrin-B in vertebrates.

### An Ancestral and Conserved Role in Cell Segregation

Ephs and ephrins follow a parallel evolutionary history to cadherins since similarly to them, the overall Eph structure found in animals was established in the metazoan ancestor by the co-option of domains (figs. 1 and 2) that together with those associated with ephrins, likely enlarged their functional capabilities compared with those in choanoflagellates. It seems clear that through their role in cell–cell contact and communication, both cadherins and Ephs/ephrins influenced the evolution of cell–cell adhesion mechanisms, contributing to the emergence of multicellularity. However, although Ephs/ephrins may be considered part of the cellular adhesome network, they play completely different roles from cadherins, including some antagonism. As such, Eph signaling downregulates cell–cell adhesion by different mechanisms, including sequestering Cdc42 (Bisson et al. 2007), cadherin cleavage (Solanas et al. 2011), or decreasing cadherin clustering (Fagotto et al. 2013). Targets of Eph/ephrin signaling include the Rho GTPases, which control actomyosin assembly and contraction. This underlies cell repulsion and antagonizes cell adhesion. Rather than differential adhesion, repulsion at tissue borders mediated by Eph-mediated tension drives cell segregation (Canty et al. 2017; Taylor et al. 2017), compatible with the main role associated with Eph/ephrin signaling in vertebrates, repulsion upon cell–cell contact. The latter has been particularly studied in the nervous system during the formation of topographic maps, neuronal migration, and axon guidance (Pasquale 2008). Indeed, the first functional analysis of Ephs and ephrins were performed in the developing nervous system in vertebrates (see, for instance, Drescher et al. 1995) becoming an extremely active field that soon established Eph/ephrin signaling as a pivotal mechanism in neural development, particularly when a role in axon guidance was also found in *Drosophila* (Dearborn et al. 2002). However, our data indicate that Eph/ephrin signaling may have occurred in the metazoan ancestor and are very likely active in Porifera, which lack a nervous system. Thus, the question that emerges is what is the ancestral role of Ephs/ephrins. Much evidence in different metazoans point to an ancestral function in the regulation of cell segregation. As such, in addition to their function in migration and axon guidance, soon after the discovery of their interaction and signaling, it was clear that vertebrate Ephs and ephrins were also involved in the segregation of cells in the paraxial mesoderm to give rise to the somites (Durbin et al. 1998) and in the hindbrain to segregate the different rhombomeres (Xu et al. 1995, 1999). Later this function was extended to the separation of ectoderm and mesoderm in *Xenopus* (Rohani et al. 2011). This role in cell segregation is conserved in nonvertebrates, as observed during the formation of the anteroposterior boundary in the *Drosophila* wing (Umetsu et al. 2014). In Cnidaria, the expression of Ephs and ephrins at tissue boundaries is also consistent with this function (Tischer et al. 2013). Importantly, cell segregation also occurs in sponges, as they react to allografts with the formation of a boundary, and are known to bear mechanisms for cellular self/nonself-recognition (Gaino et al. 1999). Thus, we propose that Ephs/ephrins influenced the evolution of adhesive mechanisms and assisted in the transition from unicellular to multicellular organisms, in parallel and counteracting cadherins to promote the segregation of cell populations, which is crucial for metazoan embryonic development.

In subsequent evolutionary steps, further Eph/ephrin signaling diversity and complexity appeared through the introduction of new intra- and extracellular effectors, parallel signaling through Ephs and ephrins coexpressed in the same cell and the expansion of the Eph and ephrin families. In addition, the appearance of splice variants and kinase-dead Ephs could introduce regulatory mechanism to attenuate Eph/ephrin signaling. Concomitantly, new functions were co-opted, including their central role in the development of the nervous system, directing migration, and axon guidance for the generation of topographic maps and circuits (Triplett and Feldheim 2012), vascular development (Adams and Eichmann 2010), cell fate decision through the control of asymmetric division (Picco et al. 2007; Franco and Carmena 2019) or endocytosis (Pitulescu and Adams 2010). Indeed, the formation of boundaries for the proper segregation of different populations remained as a fundamental role (Wilkinson 2015). In vertebrates, cross-class interactions and new lineage expansions (and losses) gave rise to the repertoire found in humans, where Eph/ephrin signaling is involved in a plethora of processes (Kania and Klein 2016; Niethamer and Bush 2019). Last, but not least, Eph deregulation has been found in disease, in particular during tumorigenesis, neurological disorders, inflammation, and repair (Batlle and Wilkinson 2012; Chen et al. 2012; Coulthard et al. 2012; Nunan et al. 2015), emerging as therapeutic targets (Boyd et al. 2014; Barquilla and Pasquale 2015). Importantly, in all these functions, a common theme is the recognition of neighboring cells through cell–cell contact to decrease cell–cell adhesion and promote cell migration and segregation.

## Materials and Methods

### Genome Sources and Sequence Retrieval

To trace the presence along evolution of orthologs of human Ephs and ephrins genes, sequence data sets for 129 proteomes were downloaded from the available databases comprising 53 prokaryotes and 76 eukaryotes (supplementary table S1, Supplementary Material online; see also, supplementary tables S2 and S3, Supplementary Material online, for Eph and ephrin sequence identifiers, respectively). These data sets include complete and incomplete proteomes, and they contain both predicted and confirmed peptide sequences. When a particular proteome was available in different databases, the version containing the highest number of human orthologs and the more accurately predicted proteins was chosen.

For those species where some proteins were missing and for those phylogenetic groups where the only genomes available are in early sequencing stages, we searched in NCBI and UniProt to try to find ephrins and Eph sequences and, when possible, to manually correct those that were incomplete or wrongly predicted.

The organisms were grouped on the basis of previously defined phylogenetic studies (Forterre 2015; Sebe-Pedros et al. 2017; Simion et al. 2017) (see fig. 2, for the phylogenetic tree of these species). The species divergence times were extracted from http://www.timetree.org/; last accessed October 2019 (Kumar et al. 2017) using consensus estimates from the literature due to the fact that dates are not available for all the species.

### Identification of Homologs/Orthologs

Homologous sequences of human proteins were identified using Inparanoid 4.1 (Remm et al. 2001), an automatic method that uses pairwise similarity scores, calculated using NCBI–Blast (Altschul et al. 1990), between two proteomes for constructing orthology clusters.

We ran the program using the default parameters except for the in-paralog confidence cutoff, which we made more stringent (from 0.05 to 0.25) to avoid obtaining too many in-paralogs with weak similarity to the main ortholog in distantly related organisms. All Inparanoid Blasts were run using a threshold e-value of 0.01 and different matrices were used in pairwise comparisons to account for different evolutionary distances: Blossum45 to compare prokaryotes, Blossum62 for eukaryotes, and Blossum80 for comparisons between metazoans.

We used HHPred (Alva et al. 2016) to detect distant sequence homology (databases selected: PDB_mmCIF70_3_jul, SCOPe70_2.06, Pfam-A_v31.0, and SMART_v6.0) and Phyre2 (Kelley et al. 2015) to predict and compare 3D protein structures.

### Protein Domain Identification

In order to analyze the domain repertoire of the whole proteomes used in this study and establish the domain emergence and domain architecture of the Eph and ephrin proteins, we ran the Hmmscan program from HMMER 3.1 (Eddy 2009, 2011) against the Pfam database (version March 31, 2017) (Finn et al. 2016). Nonoverlapping hits with scores above the conditional e-value threshold of 0.05 were considered significant. We also used hmmbuild to build profiles of the GPI-anchor, TM, and the cytoplasmic region of the ephrins and used hmmsearch to search the proteomes with them. To better determine the emergence in evolution of the domains and identify possible HGT events, NCBI Blastp (Altschul et al. 1990) searches with the domains’ consensus sequences were conducted.

### TM and GPI-Anchor Structures

Ephrin TM structures were predicted with the TMHMM Server 2.0 (http://www.cbs.dtu.dk/services/TMHMM/; last accessed October 2019) (Krogh et al. 2001) and Phobius (Kall et al. 2004), and GPI-anchor structures were predicted with big-PI Predictor (Eisenhaber et al. 1999) and PredGPI (Pierleoni et al. 2008).

### Sequence Alignment and Phylogenetic Inference

The L-INS-i model in Mafft 7.130 (Katoh and Standley 2013) was used to build a multiple sequence alignment (MSA) with the orthologous proteins of the ephrins. As we specifically wanted to know how the Ephs–ephrins interacting domains have evolved, we trimmed the Eph and ephrin protein sequences so that only the ephrin-binding domain (Ephrin_lbd) and Eph-binding domain (ephrin) remained, respectively, and used G-INS-i to build MSAs that were afterward used for the phylogenetic analyses. The alignments were visualized using Jalview 2.8 (Waterhouse et al. 2009), their quality was manually checked and they were then used as the input for ProtTest 3.4 (Abascal et al. 2005; Darriba et al. 2011) to select the model of protein evolution that best fits the set of sequences.

Finally, phylogenetic relationships were deduced using the Bayesian inference method as implemented in MrBayes 3.2.6 (Ronquist et al. 2012), performing two simultaneous runs of four Markov chains each for 5 million generations (30 million in the case of the whole Eph protein tree), sampling every 500 generations and discarding the initial 25% of the trees generated. We used the R package RWTY to assess the convergence of the analyses (Warren et al. 2017). In addition, maximum likelihood phylogenetic trees with 500 bootstrap replicates were generated with PhyML 3.1 (Guindon et al. 2010) (supplementary figs. S4 and S7, Supplementary Material online). The final trees were visualized with iTOL v3 (Letunic and Bork 2016).

### Synteny Analysis

Analysis of genomic regions was performed to confirm or clarify the homology relationship of the sequences. Genomic environments were identified in NCBI, the UCSC Genome Browser database (Tyner et al. 2017) or in the Metazome 3.2 platform (https://metazome.jgi.doe.gov/pz/portal.html; last accessed October 2019). Sequences from the tunicate *Oikopleura dioica* were searched in the OikoBase (Danks et al. 2013).

### 3D Protein Structures

3D protein structures were obtained from the Protein Data Bank (PDB) database (Burley et al. 2017), and for those proteins without a 3D structure available, 3D models were predicted with Phyre2 (Kelley et al. 2015). Structures were visualized and colored using EzMol (Reynolds et al. 2018).

### Coevolution Analyses

Coevolution of interacting Ephs and ephrins was assessed with CoeViz (Baker and Porollo 2016), which analyses coevolution of protein residues considering intraprotein residue–residue contacts using 3D structures and multiple sequence alignments, and MirrorTree (Ochoa and Pazos 2010), which analyses the coevolution of different protein families. With MirrorTree, we used a reduced set of sequences selecting one representative of each taxon except in the case of vertebrates, where we selected *P. marinus*, *Callorhinchus millii*, *Danio rerio*, *Gallus gallus*, and *Homo sapiens*. Given that both Eph and ephrin families are quite broad and they promiscuously bind many different receptors or ligands (such as VAP and others), it is quite difficult to obtain clear in silico information about their coevolution. 

## Supplementary Material

msz222_Supplementary_Material
