# Supplementary material for: The Evolutionary History of Ephs and Ephrins: Toward Multicellular Organisms
Source: Mol Biol Evol. 2019 Oct 7;37(2):379–94. doi: 10.1093/molbev/msz222 (PMC6993872; doi:10.1093/molbev/msz222)
Supplement: msz222_Supplementary_Material [file msz222_supplementary_material.pdf]

Supplementary Material

Title: The evolutionary history of Ephs and ephrins: towards multicellular organisms

Authors: Aida Arcas, David G. Wilkinson and M. Ángela Nieto

Supplementary figures S1 to S14

References for Supplementary Material

Supplementary table ST1 – Species used in this study

Supplementary table ST2 – Eph Sequence identifiers

Supplementary table ST3 – Ephrin Sequence identifiers

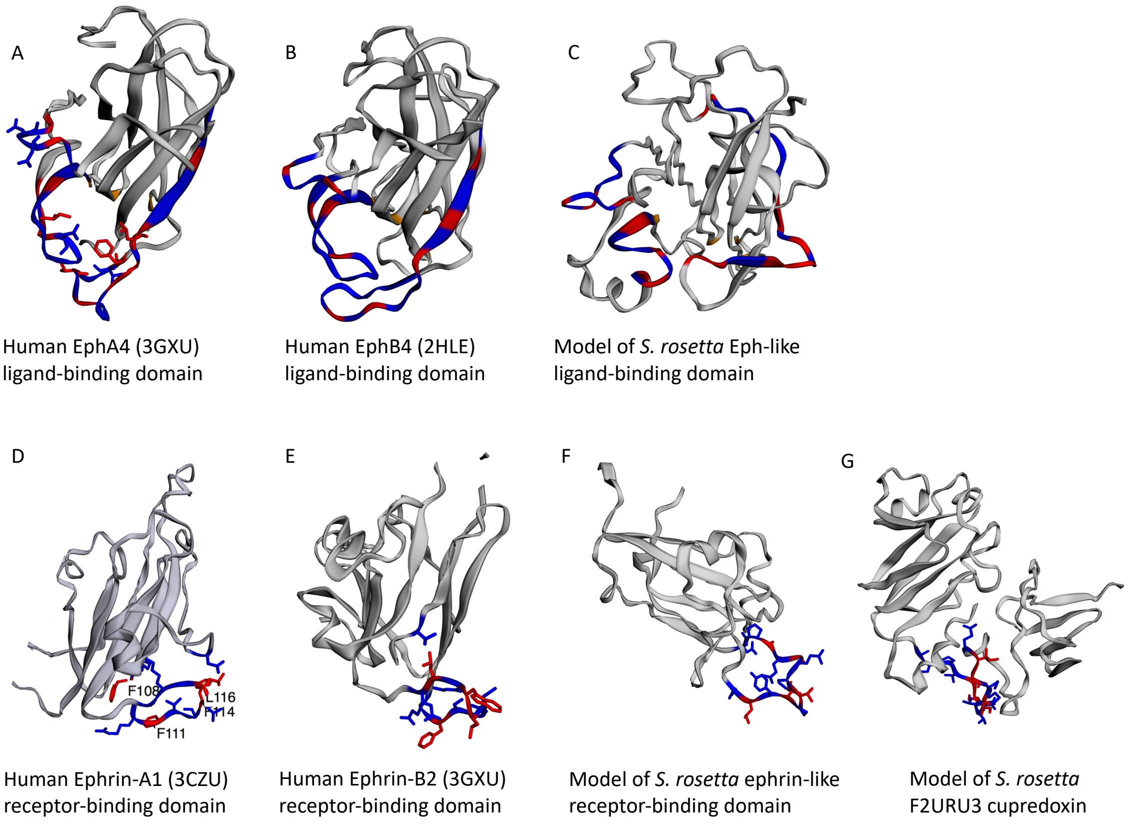

Fig. S1. 3D structures of human Eph receptors and ephrins interacting domains, and 3D model of *S. rosetta* ephrin-like and cupredoxin proteins. **(A, B)** 3D structure of the ligand-binding domain of human EphA4 and EphB4, respectively. **(C)** 3D model of *S. rosetta* ligand-binding domain in the Eph-like protein F2UER5, that forms a pocket region where the ephrin-like might bind. **(D, E)** 3D structure of the Eph-binding domain of human ephrin-A1 and ephrin-B2, respectively. **(F)** 3D model of *S. rosetta* receptor-binding domain in the ephrin-like protein F2TW21, that has a protruding loop that could interact with the Eph-like pocket similarly to metazoan ephrins **(G)** 3D model of *S. rosetta* cupredoxin F2URU3, whose fold is completely different from the ephrin-like sequence (F) and the equivalent residues are not exposed in the cupredoxin. Human 3D structures (in interacting conformation) were obtained from the PDB database (Burley et al. 2017). Residues in the interacting Eph-ephrin surface (Himanen et al. 2010; Qin et al. 2010) are coloured blue, and among them, the hydrophobic residues are highlighted in red. The cysteines that form the disulphide bridges in the Ephs' ligand-binding domain are marked in orange. The equivalent residues in the choanoflagellate's sequences have been coloured accordingly.

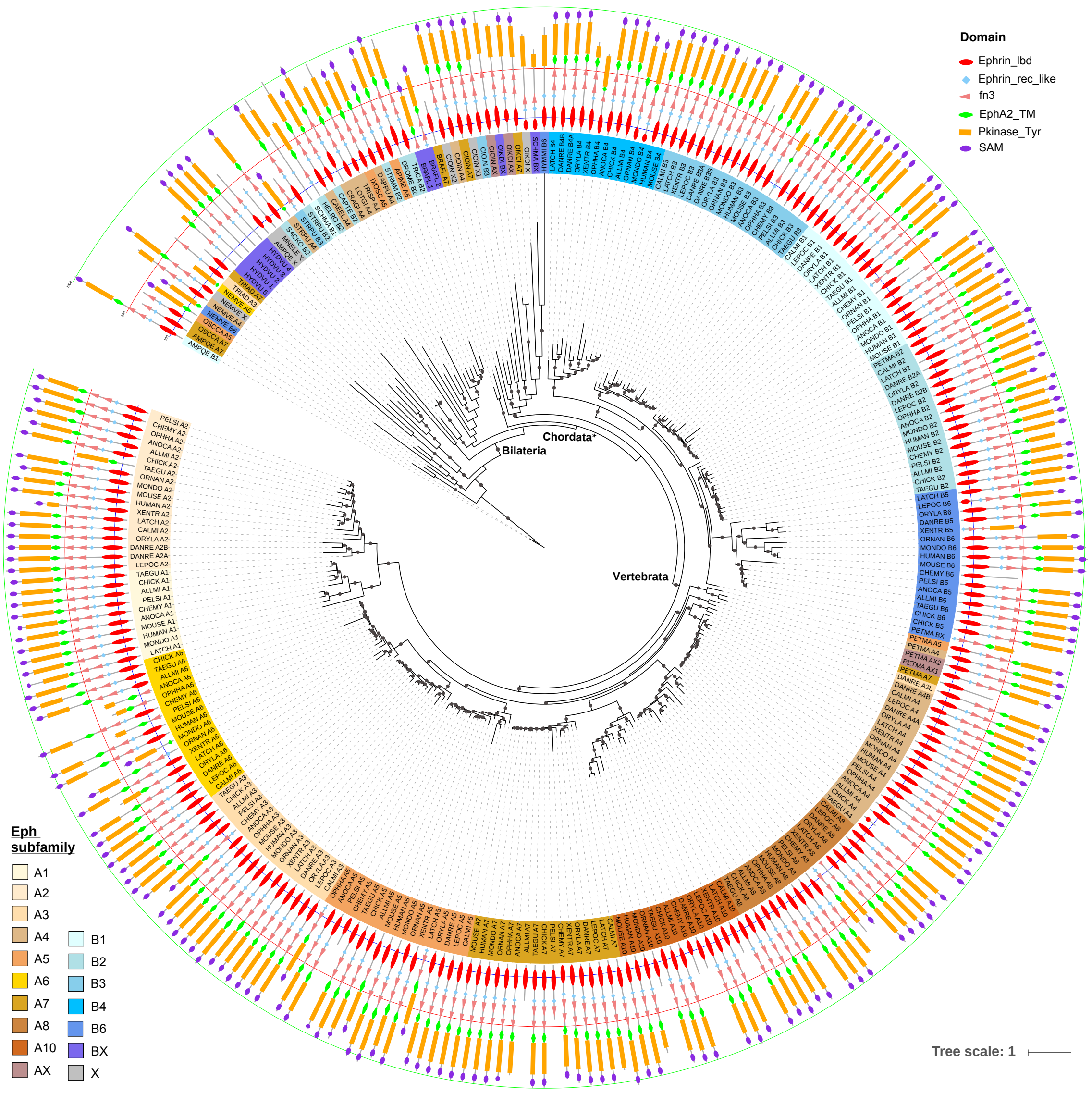

Fig. S2. Phylogenetic tree of Eph proteins reconstructed by Bayesian inference (BI). Model of amino acid evolution: JTT+G. 280 sequences from 44 species are included in the tree. Black circles at nodes indicate clades with posterior probability > 70%. Branches are drawn proportional to the number of amino acid substitutions per site as indicated by the scale bar (lower right). The legend on the top right depicts the domains as defined by Pfam. Although this tree did not reach convergence after 30 million generations, species group according to the tree of life except for two bilaterian sequences from the platyhelminthes *S. mansoni* (SCHMA) and *H. microstoma* (HYMMI) that group with chordates (asterisk). Ephs are clearly separated into class-A and -B only in vertebrates. See supplementary tables ST1-ST3 for information on the species and sequences.

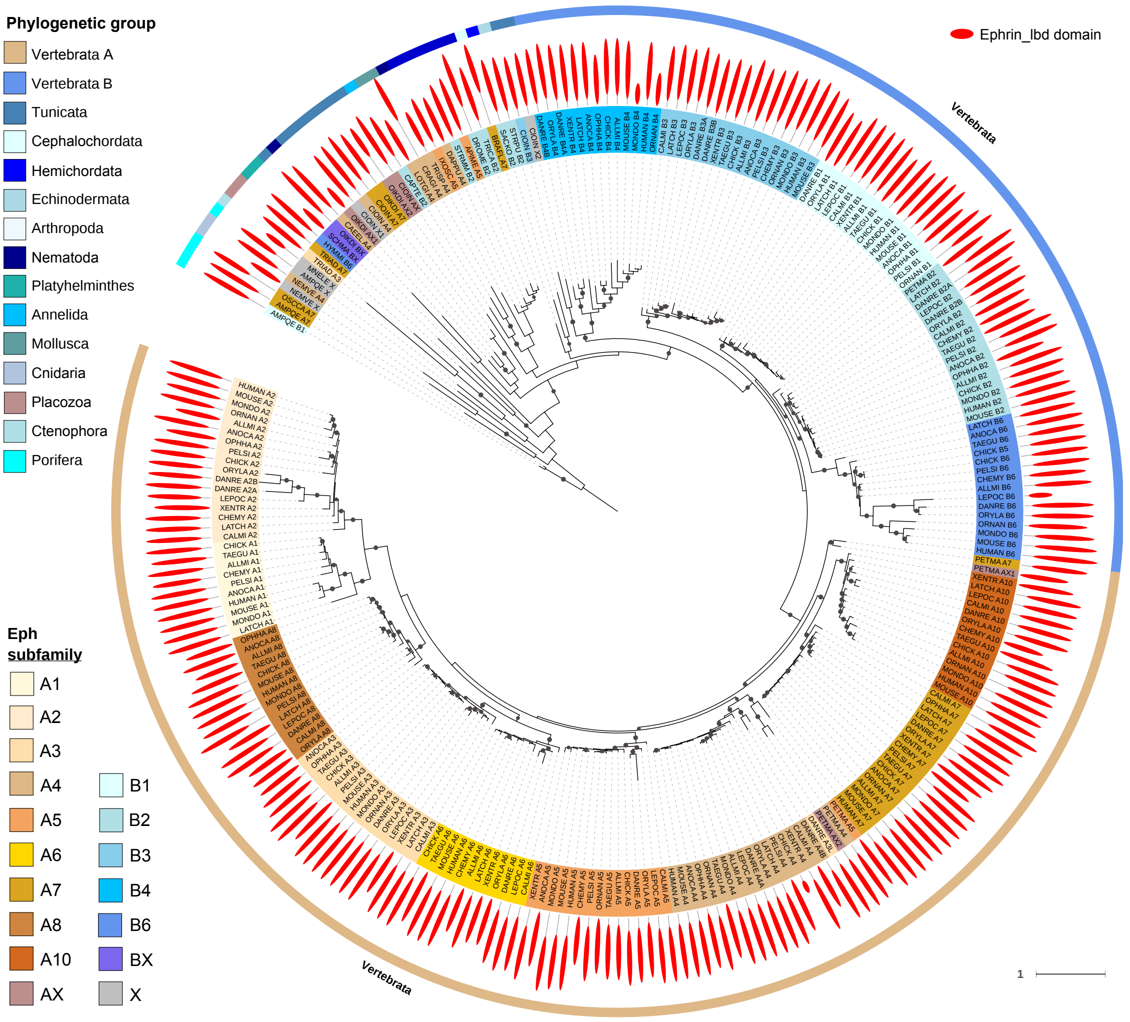

Fig. S3. Phylogenetic tree of the ephrin-binding domain (Ephrin_lbd) in Ephs reconstructed by BI. Model of amino acid evolution: JTT+I+G. 251 sequences from 41 species are included in the tree. Ephs are clearly separated into class-A and -B only in vertebrates. The sequences are annotated as predicted by InParanoid and have been coloured accordingly; we have included into subfamilies AX or BX those sequences whose best hits were to class-A or class-B Ephs, respectively, although they were not unequivocally assigned to a particular orthologue. Subfamily X includes sequences whose best hits did not differentiate class-A and -B human Ephs. The ephrin-binding domain (Ephrin_lbd) in each sequence is always indicated (see legend on top right) although it was incomplete for some proteins (e.g. Chick_A4 or Mondo_B4). The outer strip colour marks the different phyla (top left). Black circles at nodes indicate clades with posterior probability > 70%. Branches are drawn proportional to the number of amino acid substitutions per site as indicated by the scale bar (lower right). The topology of this tree is very similar to that built with the full Eph protein sequences (fig. S2). The most notable difference is the position of EphA4 as the most ancient type-A Eph receptor in the latter, which might be due to the distinct rate of evolution of different domains (Wolf et al. 2008; Mikami et al. 2012). See supplementary tables ST1-ST3 for information on the species and sequences.

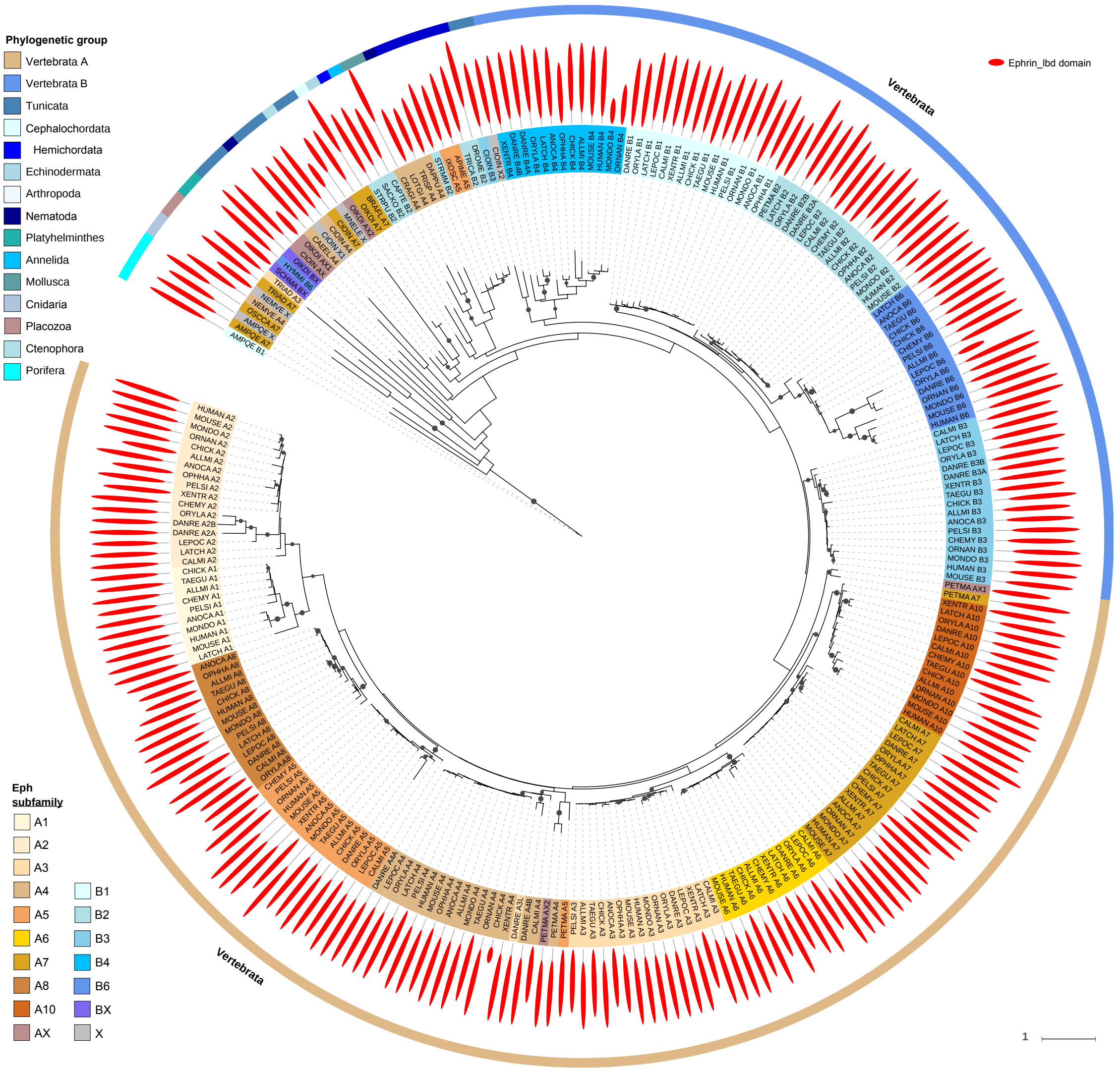

Fig. S4. Phylogenetic tree of the ephrin-binding (Ephrin_lbd) domain in Ephs reconstructed by Maximum Likelihood. This tree is very similar to that built by BI (fig. S3), with slight differences. For example, *P. marinus* EphAs are less close to the EphAs sequences sub-branch root than in that constructed by BI, and EphB3 is farther away from EphB4 sequences in this tree. See supplementary tables ST1-ST3 for information on the species and sequences.

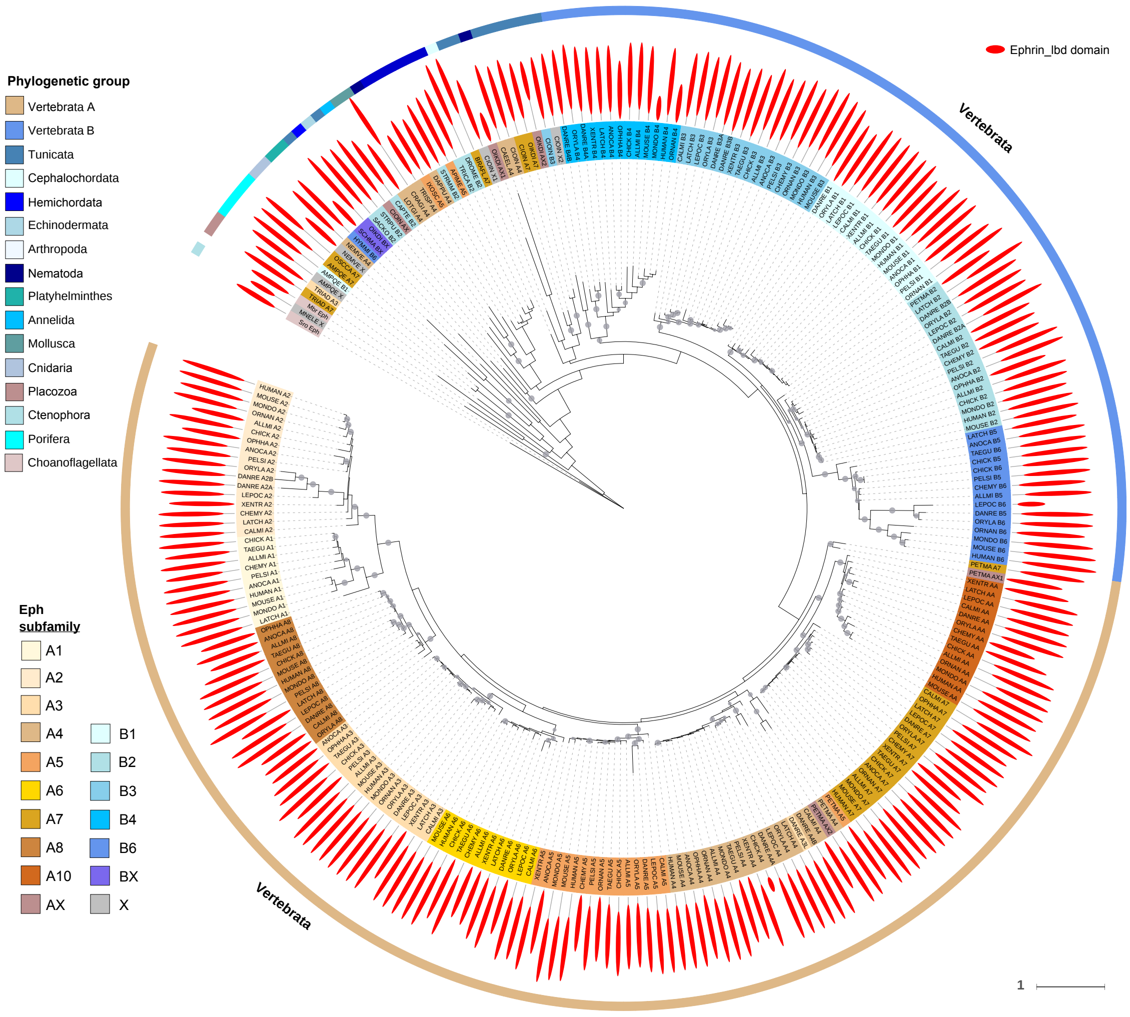

Fig. S5. Phylogenetic tree of the ephrin-binding domain (Ephrin_lbd) in Ephs reconstructed by BI and rooted with *S. rosetta* Eph-like protein. Model of amino acid evolution: JTT+I+G. 253 sequences from 43 species are included in the tree. Including the *S. rosetta* and *M. brevicollis* Eph-like proteins barely alters the topology of the tree. Compared to fig. S3, now Ctenophora and Placozoa group closer to the root than poriferan sequences, and all tunicate sequences group closer to vertebrate. The rest of the tree follows quite well the species taxonomy and the position of vertebrates and tunicates is identical as in the tree built without these choanoflagellate sequences. See supplementary tables ST1-ST3 for information on the species and sequences.

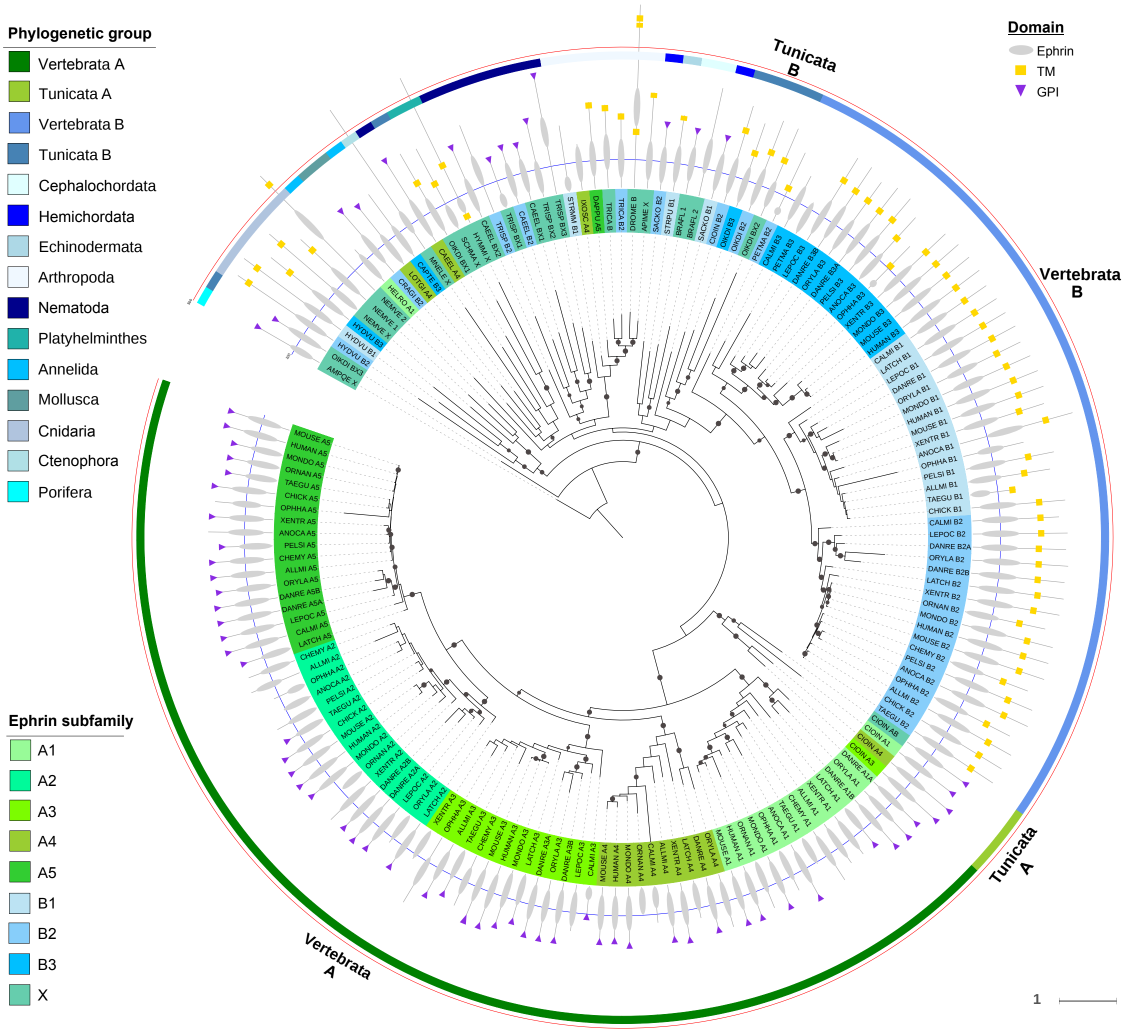

Fig. S6. Phylogenetic tree of ephrins reconstructed by BI. Model of amino acid evolution: JTT+I+G. 164 sequences from 41 species are included in the tree. Vertebrates and tunicates class-A ephrins have a GPI-anchor in the C-terminal region of the protein, while class-B ephrins contain a TM region. In other phyla, ephrins cannot be easily classified into class A and B, as different groups have GPI-anchor or TM regions that seem to have been shuffled along evolution. Sequences are annotated as predicted by InParanoid and have been coloured accordingly; we have included into subfamily X those sequences which best hits were to both class-A and -B human ephrins. The outer strip colour marks the different phyla (top left). For information on the domain architecture of the different sequences see legend on top right corner. Black circles at nodes indicate clades with posterior probability > 70%. Branches are drawn proportional to the number of amino acid substitutions per site as indicated by the scale bar (lower right). See supplementary tables ST1-ST3 for information on the species and sequences.

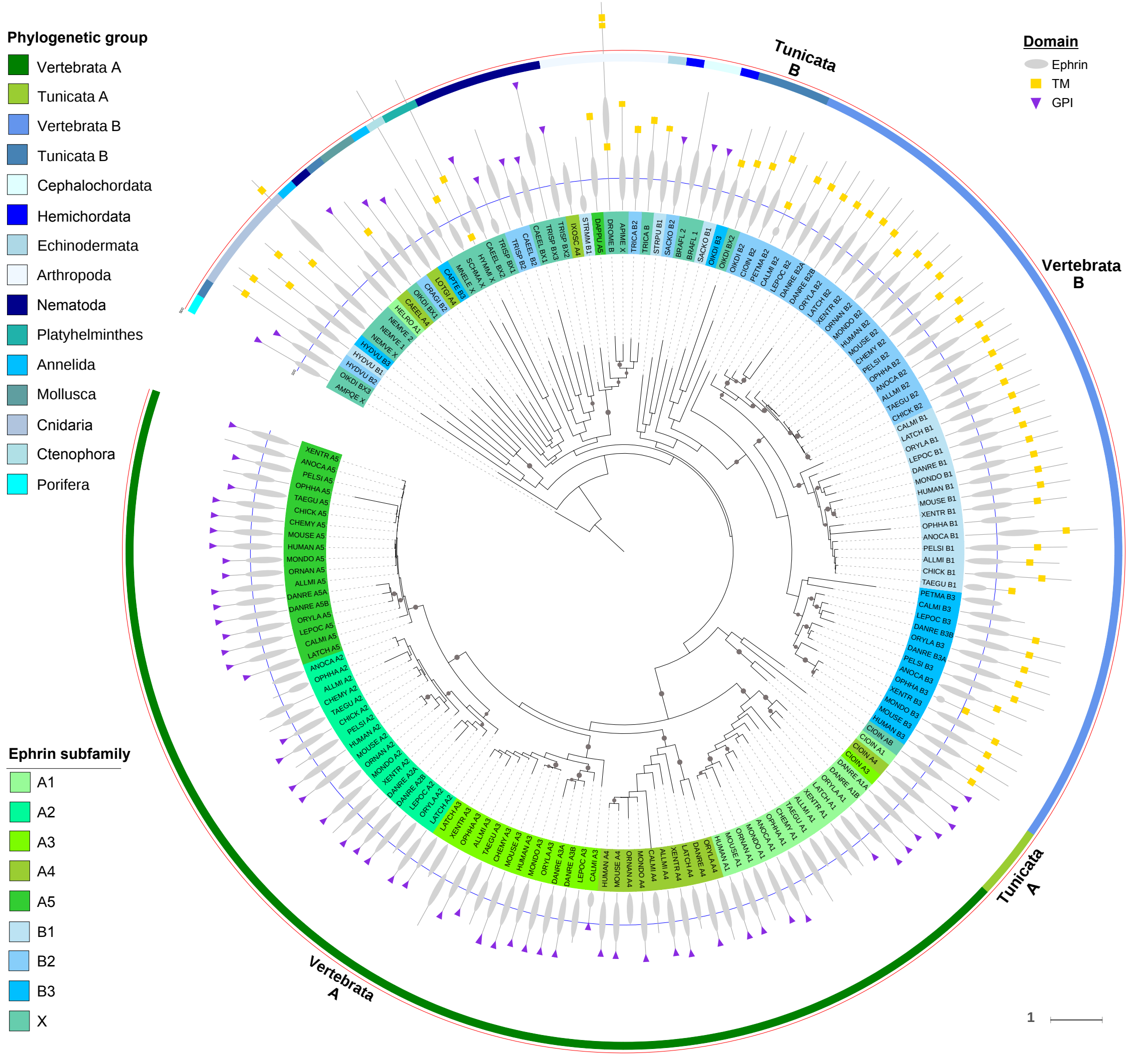

Fig. S7. Phylogenetic tree of ephrins reconstructed by ML. This tree is very similar to that obtained by BI (fig. S6), the main difference being that Deuterostomia sequences are less close to vertebrate class-B ephrins in this tree. See supplementary tables ST1-ST3 for information on the species and sequences.

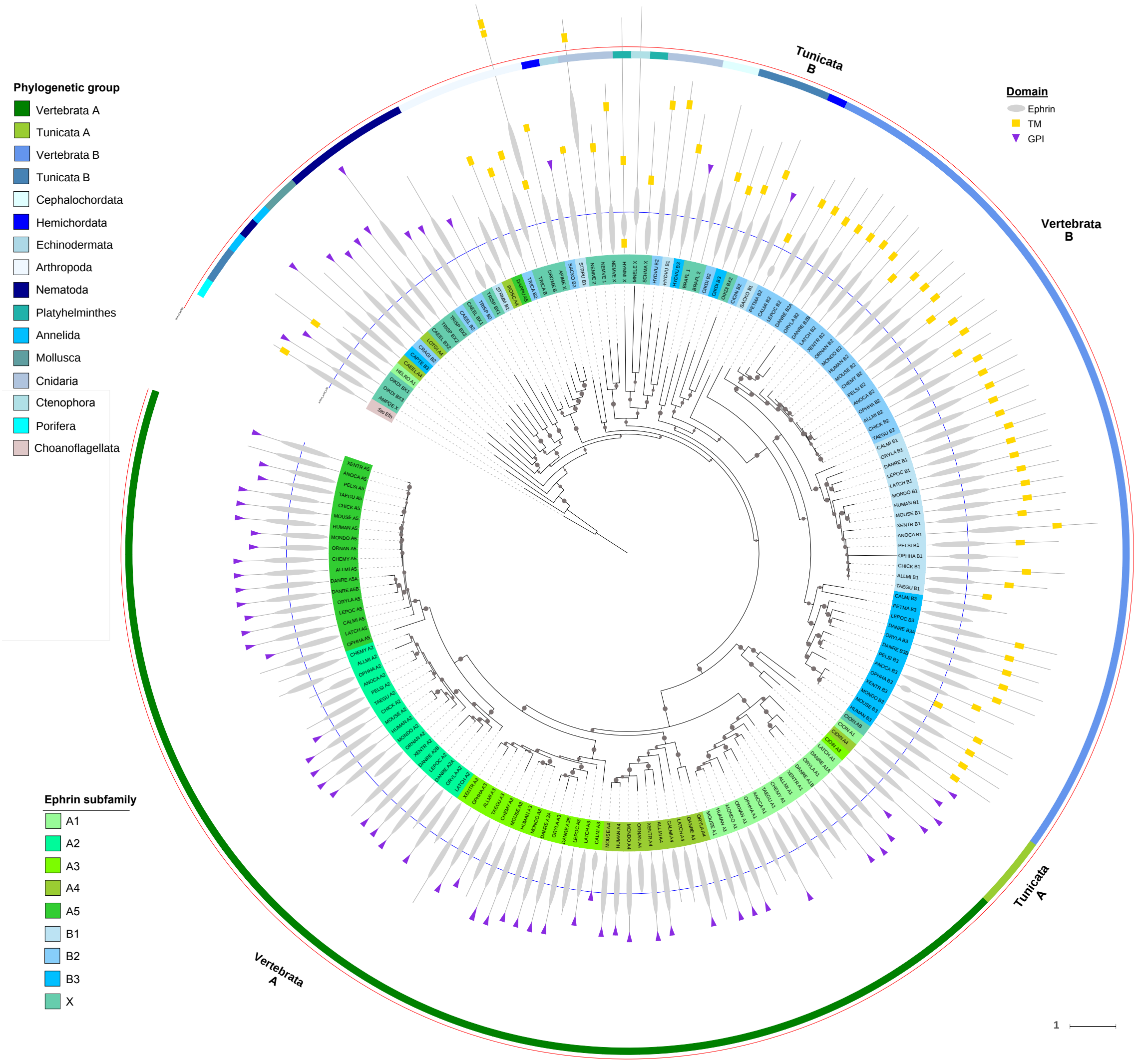

Fig. S8. Phylogenetic tree of ephrins reconstructed by BI and rooted with *S. rosetta* ephrin-like protein. Model of amino acid evolution: JTT+I+G. 165 sequences from 42 species are included in the tree. Compared to fig. S6, now cnidarian sequences group further away from the root and it seems that the organisms are sorted less like in the taxonomic tree. Nevertheless, the position of vertebrates and tunicates is identical as in the tree built without these choanoflagellate sequences. Overall, when rooting the trees with the putative choanoflagellate orthologs the topology changes slightly, and these changes are mainly in nodes with low posterior probability values. See supplementary tables ST1-ST3 for information on the species and sequences.

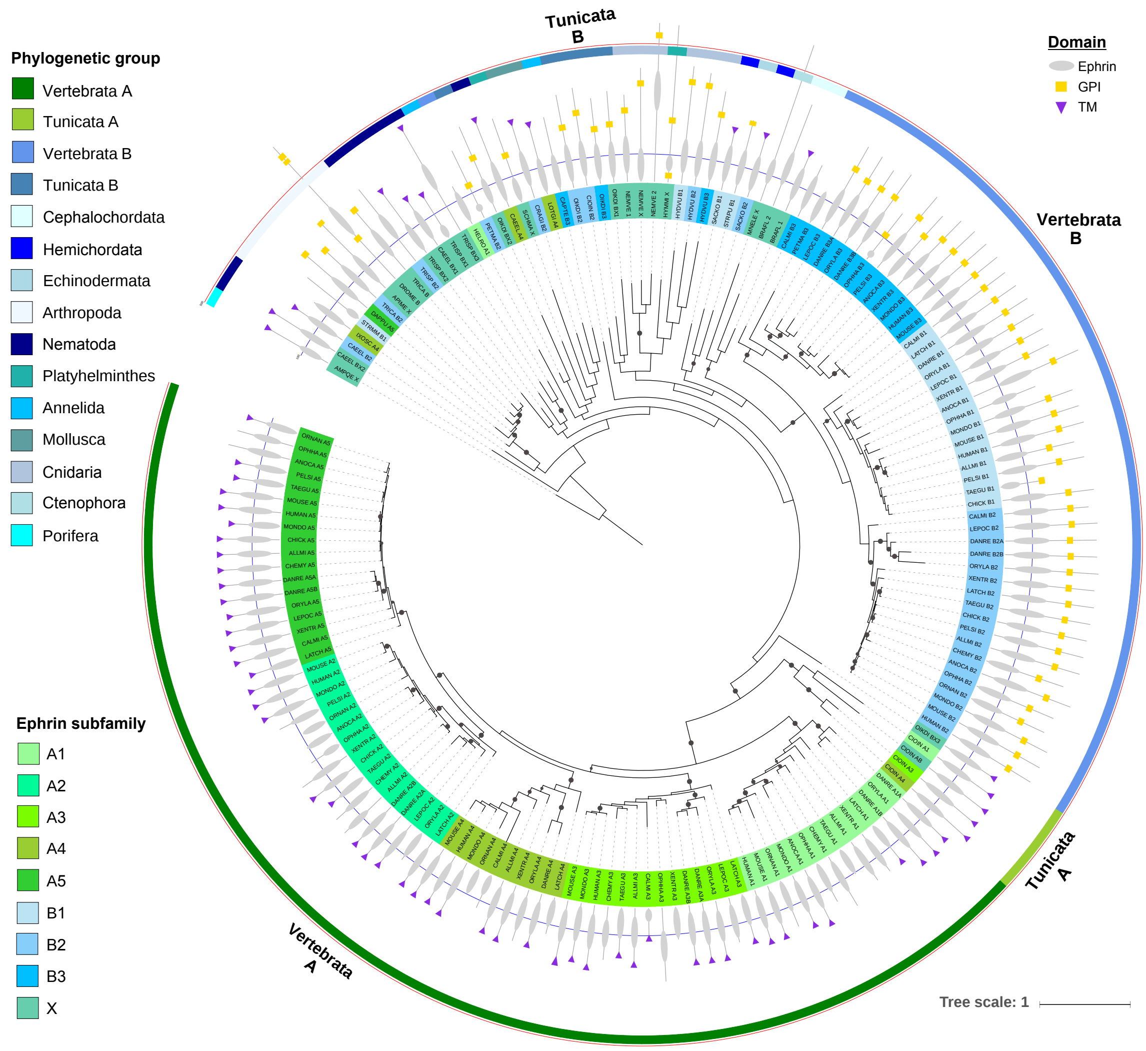

Fig. S9. Phylogenetic tree of the Eph-binding (Ephrin) domain in ephrins reconstructed by BI. Model of amino acid evolution: LG+I+G. 164 sequences from 41 species are included in the tree. Although only the Eph-binding domain has been used, the whole protein domain content has been depicted to show the distribution of membrane anchoring domains. This tree is very similar to that obtained with the whole ephrin protein sequences. See supplementary tables ST1-ST3 for information on the species and sequences.

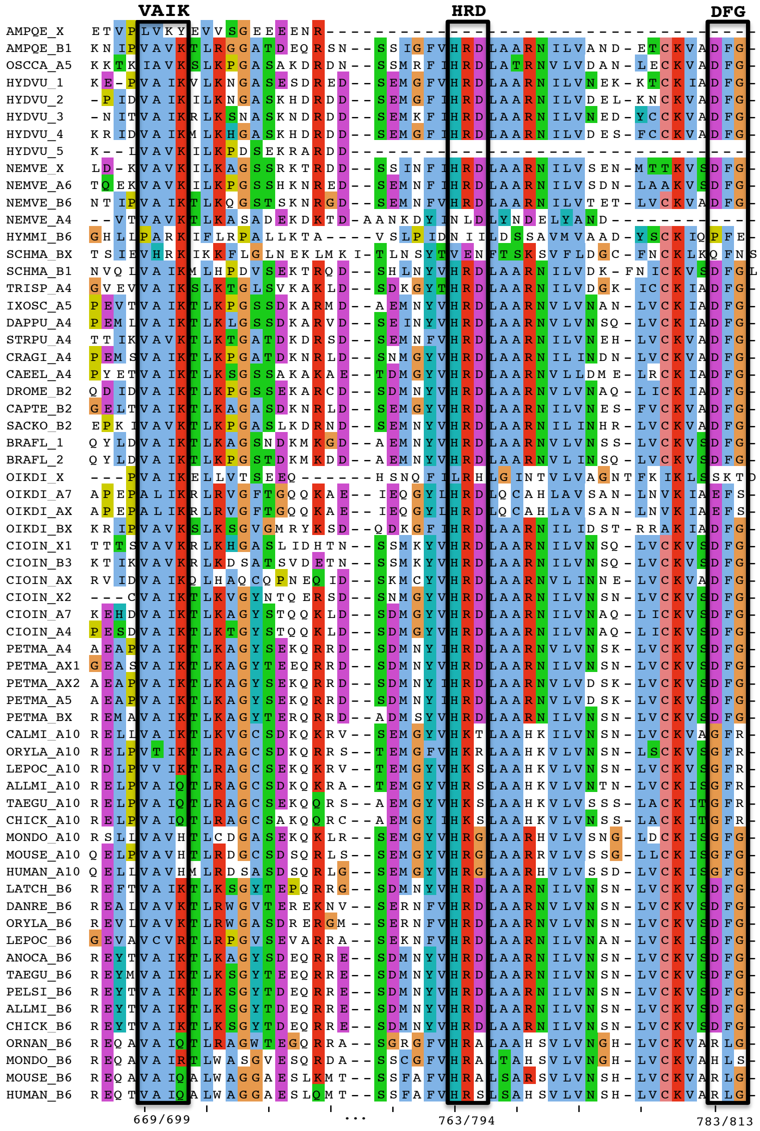

Fig. S10. Conservation of the VAIK, HRD and DFG motifs in the kinase domain of Eph orthologues. *A. queenslandica* (Porifera) has a valine instead of Isoleucine in the VAIK motif, although this should not alter its kinase catalytic capabilities since this amino acid change also appears in some Ephs of cnidarians, echinoderms, hemichordates, tunicates and in active Ephs in vertebrates. *H. vulgaris* (Cnidaria) is the most ancient organism where the three motifs are conserved in all Ephs except in a truncated one. In tunicates, the motifs are conserved in all Ephs of *C. intestinalis*, but only in one of *O. dioica*, which could be related to the differences we observed in the ephrins from these two species, and to the fast evolving genome of *O. dioika* (Berna and Alvarez-Valin 2014). Regarding vertebrates, all EphA10 orthologues are kinase-dead. We do not know whether the modified residues already appeared in Agnatha due to the available sequences from *P. marinus*. EphB6 was kinase-active when it emerged, but lost its catalytic activity in a mammalian ancestor. The numbers at the bottom respectively refer to the position of the first amino acid in the motif in Human EphA10 and EphB6 sequences, and the three dots indicate part of the sequence which is not shown. See supplementary tables ST1-ST3 for information on the species and sequences.

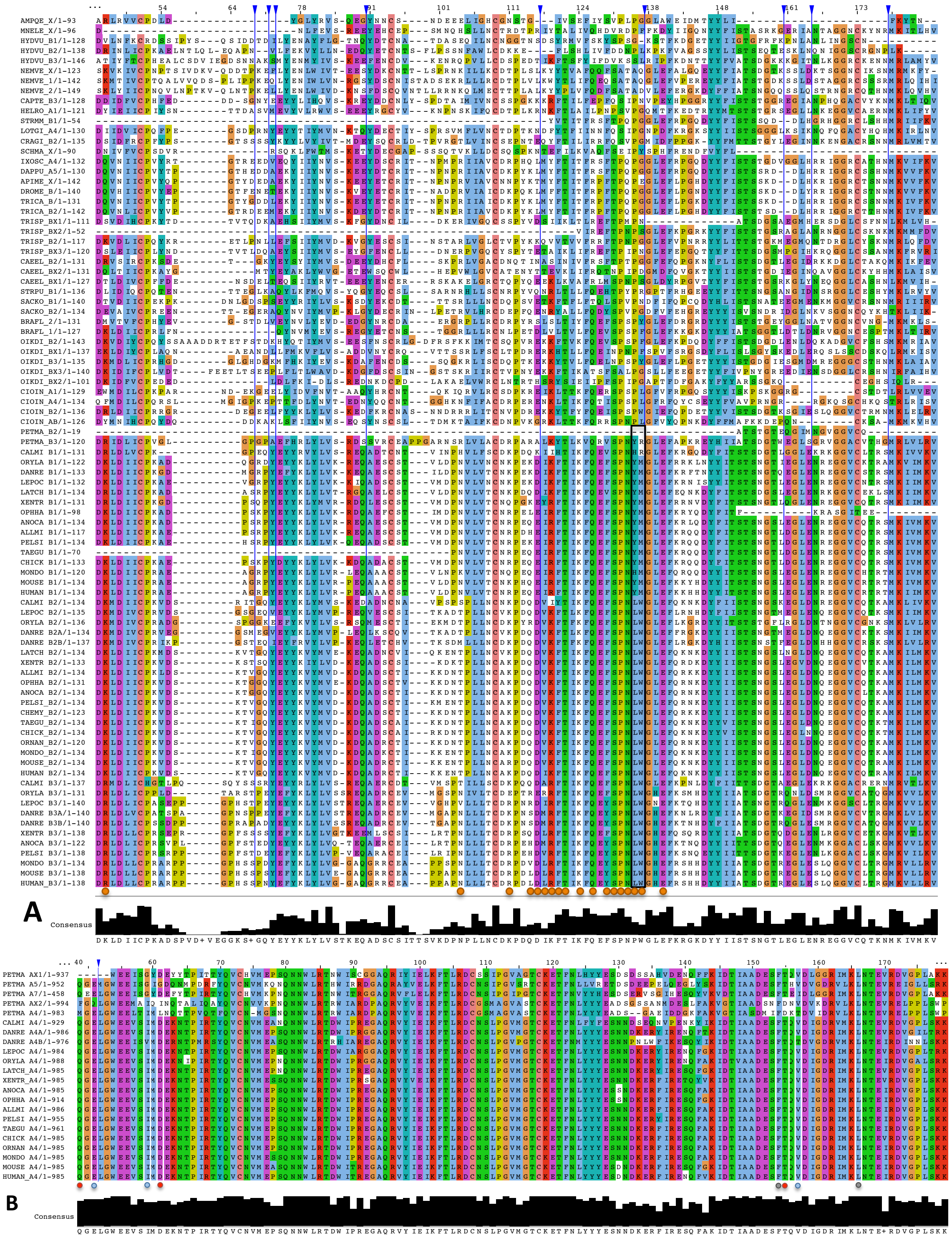

Fig S11. Conservation of residues involved in EphA4/ephrin-B2 and -B3 cross-class binding. **(A)** Multiple sequence alignment of invertebrate ephrins and vertebrate ephrin-B orthologues showing the region involved in the binding of ephrin-B2 and B3 to EphA4. The Leu and Trp residues required for the binding to EphA4 (within a black frame) are present in all Gnathostomata. *P. marinus* ephrin-B3 lacks these residues and its ephrin-B2 orthologue is a fragment that lacks the region of interaction. Blue inverted triangles mark hidden columns in the alignment. Orange dots at the bottom of the aligned columns mark the residues involved in the Eph/ephrin binding.

(B) Multiple sequence alignment of EphA4 orthologues and *P. marinus* EphAs showing the pocket region involved in the binding of EphA4 to ephrin-Bs. All residue numbers correspond to the positions in human EphA4. Dots at the bottom of the aligned columns mark those residues involved in the Eph/ephrin binding, and the dot colour indicates that the residue is conserved in: all sequences (grey), all orthologues (blue), or not conserved in all orthologues (red). The *P. marinus* sequences do not contain all residues described as necessary for cross-class interaction in human. See supplementary tables ST1-ST3 for information on the species and sequences.

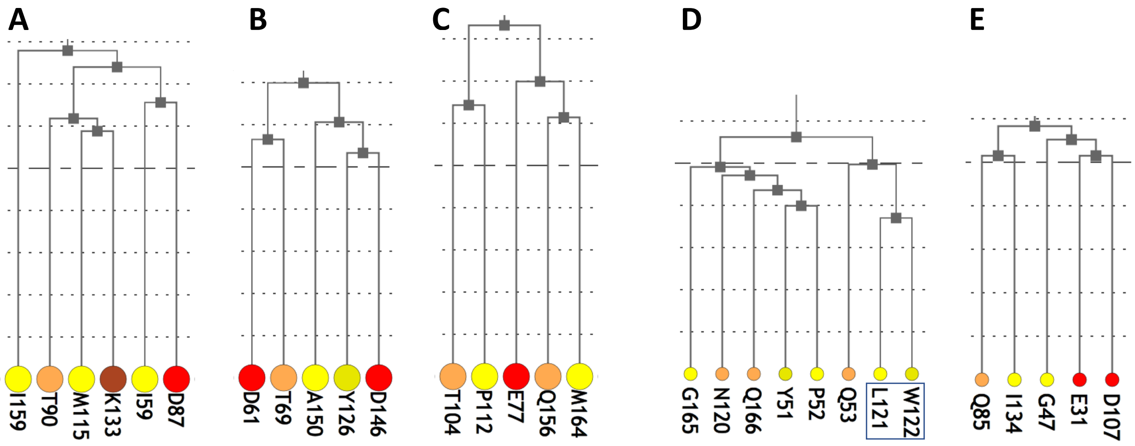

Fig S12. Co-evolving residues involved in EphA4/ephrin-B2 and -B3 cross-class binding in Gnathostomata. **(A to C)** clusters of co-evolving EphA4 residues that interact with ephrin-B2 and -B3. **(D and E)** clusters of co-evolving ephrin-B2 residues that interact with EphA4. We used CoeViz with the 2WO2 (human EphA4-ephrinB2 complex) PDB structure to analyse the conservation and changes of pairs and clusters of residues involved in the EphA4-ephrinB2 cross-class interaction. The main interacting residues in EphA4 are Q40, E42, I59, D61, F154, T155, V157 and L166 (see supplementary fig. S11B). *P. marinus* S4RX53, the Eph receptor with a higher conservation of these residues, shows differences compared to gnathostomatan EphA4: I59G, D61G and Q156H. According to CoeViz, these residues co-evolve with other residues in the protein (fig. S12A-C). Besides the I59G change in *P. marinus*, in the co-evolving cluster D87 is H in the lamprey (fig. S12A), in the D61 cluster, another two residues change in *P. marinus*: T69F and Y126V (fig. S12) and finally in the Q156 cluster, M164 is V and E77 is K (fig. S12C). Regarding ephrin-B2, the most important residues involved in the interaction with EphA4, L121 and W122 (within dark blue frame in the figure), are not conserved in *P. marinus* (supplementary fig. S11A). However, the predicted co-evolving residues (fig. S12D) are all conserved in the lamprey (supplementary fig. S11A). Among the other residues involved in this interaction (supplementary fig. S11A), the only change is D107A. D107 is in a cluster with four residues that are all conserved in *P. marinus* except Q85S (fig. S12E).

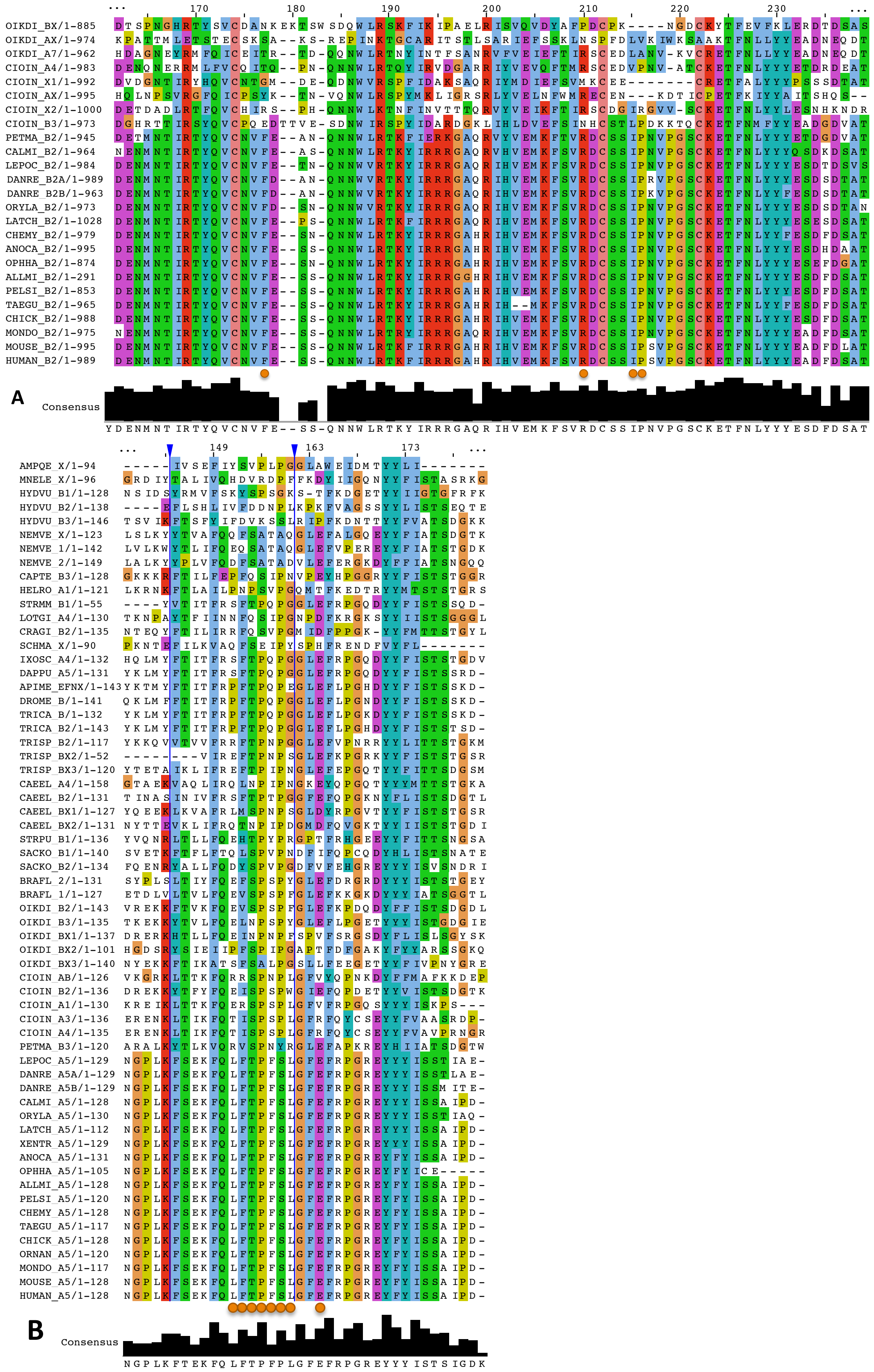

Fig. S13. Conservation of residues involved in EphB2/ephrin-A5 cross-class binding.

(A) Multiple sequence alignment of EphB2 orthologues and EphB-like sequences from invertebrates. Orange dots at the bottom of the aligned columns mark those residues involved in the EphB2/ephrin-A5 binding (Himanen et al. 2004) in human (Phe65, Arg95, Ile100 and Pro101). These residues are conserved in all vertebrate orthologues. **(B)** Multiple sequence alignment of ephrinA5 orthologues and ephrin sequences from invertebrates. Orange dots at the bottom of the aligned columns mark the residues involved in the cross-class Eph/ephrin binding in human (Leu120 to Leu126, and Glu129). These residues are only conserved in Gnathostomata. Blue inverted triangles mark hidden columns in the alignment. See supplementary tables ST1-ST3 for information on the species and sequences.

A **EphA forward signalling regulation of cell repulsion/segregation**

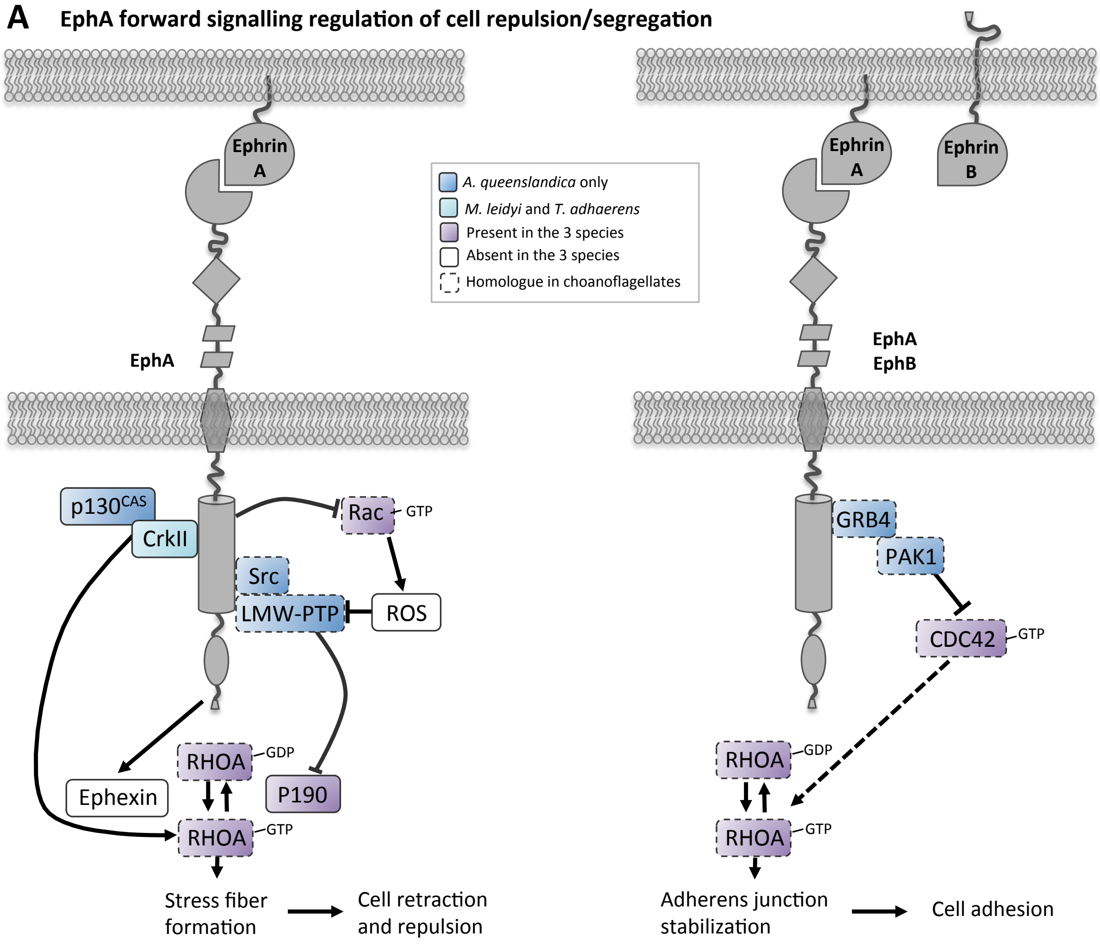

B **EphB forward signalling regulating cell-cell repulsion/segregation**

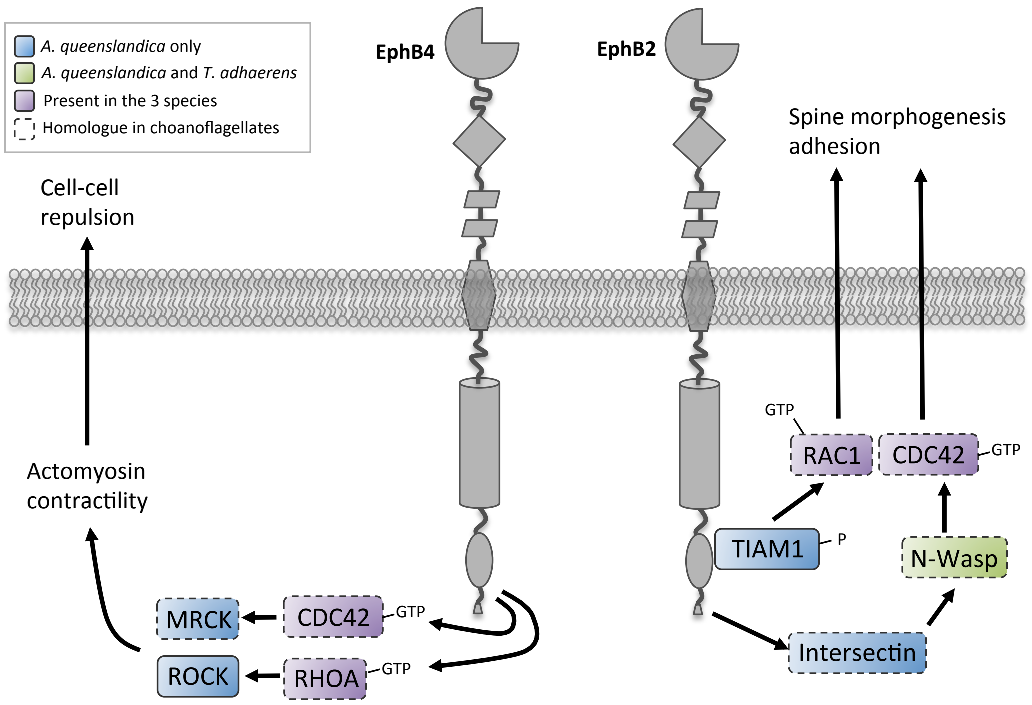

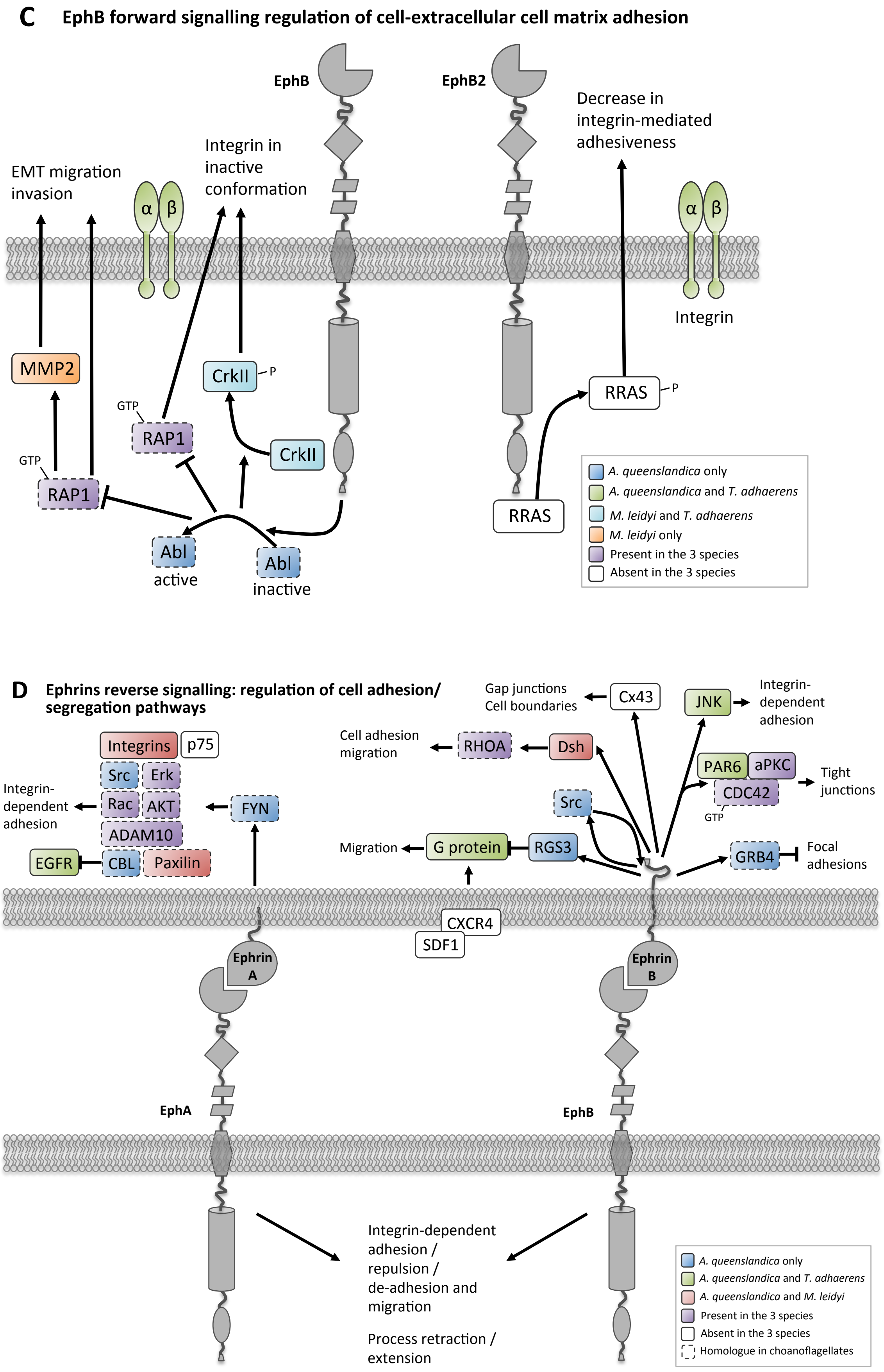
C EphB forward signalling regulation of cell-extracellular cell matrix adhesion
EphB
EphB2
Decrease in integrin-mediated adhesiveness
Integrin in inactive conformation
EMT migration invasion
α
β
Integrin
MMP2
CrkII
P
RRAS
P
GTP
RAP1
CrkII
GTP
RAP1
Abl
active
Abl
inactive
RRAS
A. queenslandica only
A. queenslandica and T. adhaerens
M. leidyi and T. adhaerens
M. leidyi only
Present in the 3 species
Absent in the 3 species
D Ephrins reverse signalling: regulation of cell adhesion/ segregation pathways
Gap junctions
Cell boundaries
Cx43
JNK
Integrin-dependent adhesion
Integrins
p75
Cell adhesion migration
RHOA
Dsh
PAR6
aPKC
Tight junctions
Src
Erk
CDC42
GTP
Integrin-dependent adhesion
Rac
AKT
FYN
Src
ADAM10
Migration
G protein
RGS3
GRB4
Focal adhesions
EGFR
CBL
Paxilin
CXCR4
SDF1
Ephrin A
Ephrin B
EphA
EphB
Integrin-dependent adhesion / repulsion / de-adhesion and migration
Process retraction / extension
A. queenslandica only
A. queenslandica and T. adhaerens
A. queenslandica and M. leidyi
Present in the 3 species
Absent in the 3 species
Homologue in choanoflagellates

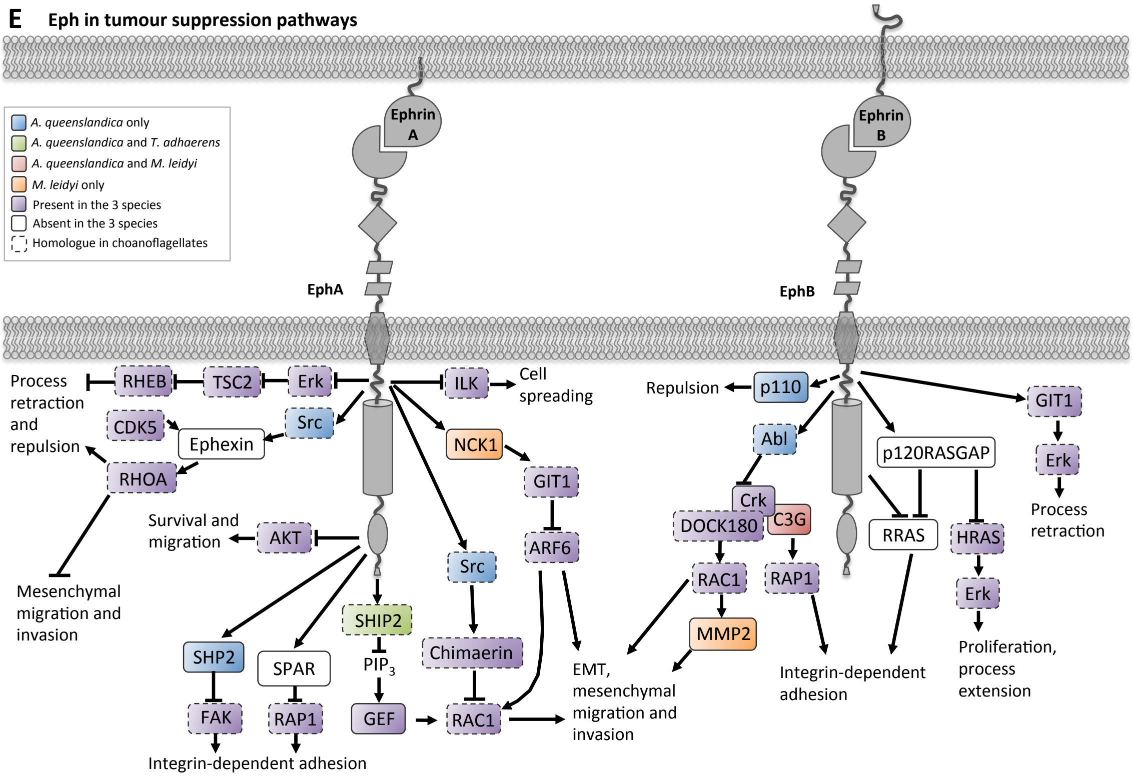
E
Eph in tumour suppression pathways
A. queenslandica only
A. queenslandica and T. adhaerens
A. queenslandica and M. leidyi
M. leidyi only
Present in the 3 species
Absent in the 3 species
Homologue in choanoflagellates
Ephrin A
Ephrin B
EphA
EphB
Process retraction and repulsion
RHEB
TSC2
Erk
ILK
Cell spreading
CDK5
Src
Ephexin
NCK1
RHOA
GIT1
Survival and migration
AKT
ARF6
Src
Mesenchymal migration and invasion
SHIP2
SHP2
SPAR
PIP3
Chimaerin
FAK
RAP1
GEF
RAC1
Integrin-dependent adhesion
EMT, mesenchymal migration and invasion
Repulsion
p110
GIT1
Abl
p120RASGAP
Erk
Crk
Process retraction
DOCK180
C3G
RRAS
HRAS
RAC1
RAP1
Erk
MMP2
Integrin-dependent adhesion
Proliferation, process extension

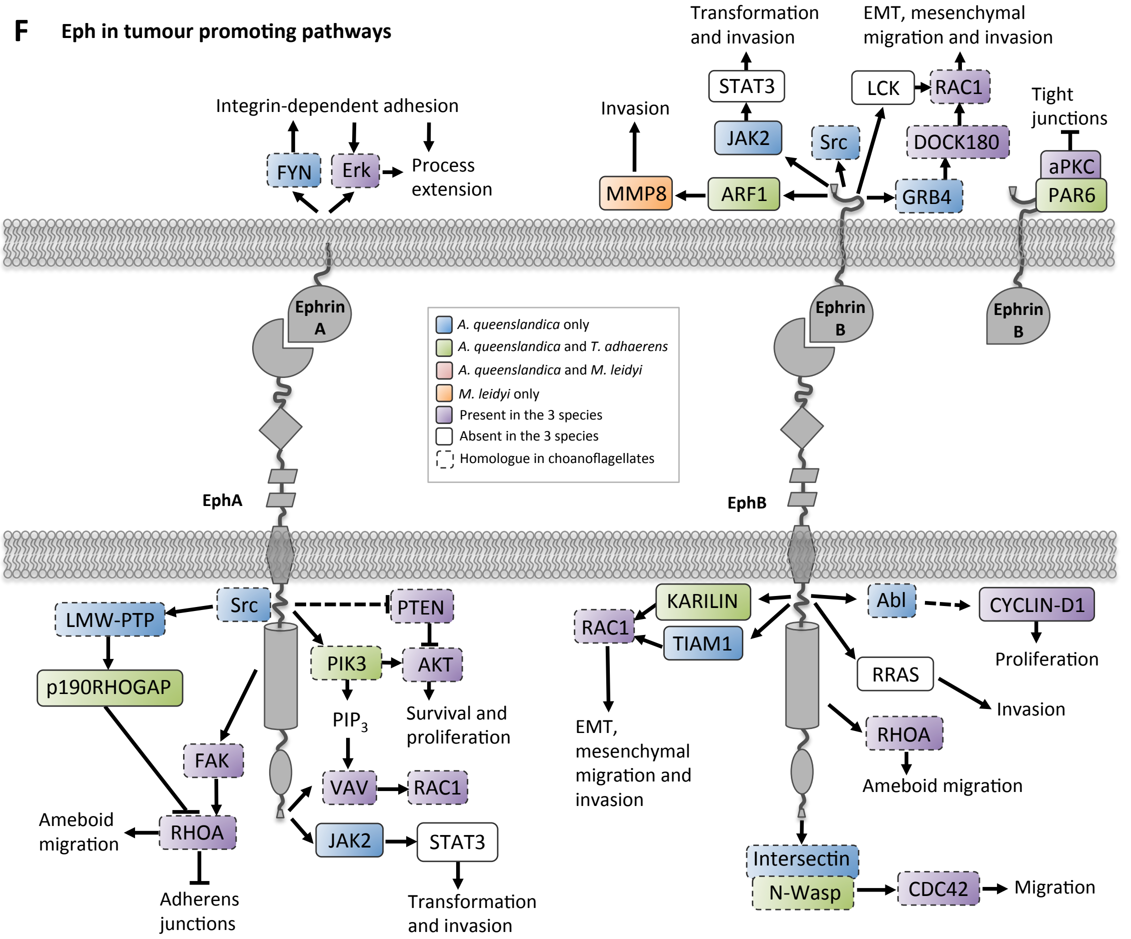
F
Eph in tumour promoting pathways
Integrin-dependent adhesion
FYN
Erk
Process extension
Invasion
MMP8
ARF1
Transformation and invasion
STAT3
JAK2
Src
EMT, mesenchymal migration and invasion
LCK
RAC1
DOCK180
GRB4
Tight junctions
aPKC
PAR6
Ephrin A
Ephrin B
Ephrin B
A. queenslandica only
A. queenslandica and T. adhaerens
A. queenslandica and M. leidyi
M. leidyi only
Present in the 3 species
Absent in the 3 species
Homologue in choanoflagellates
EphA
EphB
LMW-PTP
Src
PTEN
p190RHOGAP
PIK3
AKT
PIP3
Survival and proliferation
FAK
VAV
RAC1
Ameboid migration
RHOA
JAK2
STAT3
Adherens junctions
Transformation and invasion
KARILIN
Abl
CYCLIN-D1
RAC1
TIAM1
Proliferation
RRAS
Invasion
EMT, mesenchymal migration and invasion
RHOA
Ameboid migration
Intersectin
N-Wasp
CDC42
Migration

Fig. S14. Eph/ephrin signalling pathways. **(A)** EphA forward signalling regulation of cell repulsion/segregation. **(B)** EphB forward signalling regulating cell-cell repulsion/ segregation. **(C)** EphB forward signalling regulation of cell-extracellular cell matrix adhesion. **(D)** Ephrins reverse signalling: regulation of cell adhesion/segregation pathways. **(E)** Eph in tumour suppression pathways. **(F)** Eph in tumour promoting pathways. Ephs pathways (A to D) adapted from (Singh et al. 2012) or (Pasquale 2010) (E, F).

References for supplementary material

Berna L, Alvarez-Valin F. 2014. Evolutionary genomics of fast evolving tunicates. Genome Biol Evol 6:1724-1738.

Burley SK, Berman HM, Kleywegt GJ, Markley JL, Nakamura H, Velankar S. 2017. Protein Data Bank (PDB): The Single Global Macromolecular Structure Archive. Methods Mol Biol. 1607:627-641.

Himanen JP, Chumley MJ, Lackmann M, Li C, Barton WA, Jeffrey PD, Vearing C, Geleick D, Feldheim DA, Boyd AW, et al. 2004. Repelling class discrimination: ephrin-A5 binds to and activates EphB2 receptor signaling. Nat Neurosci 7:501-509.

Himanen JP, Yermekbayeva L, Janes PW, Walker JR, Xu K, Atapattu L, Rajashankar KR, Mensinga A, Lackmann M, Nikolov DB, Dhe-Paganon S. 2010. Architecture of Eph receptor clusters. Proc Natl Acad Sci USA. 107:10860-5.

Kelley LA, Mezulis S, Yates CM, Wass MN, Sternberg MJ. 2015. The Phyre2 web portal for protein modeling, prediction and analysis. Nat Protoc 10:845-858.

Mikami T, Miyashita H, Takatsuka S, Kuroki Y, Matsushima N. 2012. Molecular evolution of vertebrate Toll-like receptors: evolutionary rate difference between their leucine-rich repeats and their TIR domains. Gene 503:235-43.

Pasquale EB. 2010. Eph receptors and ephrins in cancer: bidirectional signalling and beyond. Nat Rev Cancer 10:165-180.

Qin H, Noberini R, Huan X, Shi J, Pasquale EB, Song J. 2010. Structural characterization of the EphA4-Ephrin-B2 complex reveals new features enabling Eph-ephrin binding promiscuity. J Biol Chem. 285:644-54

Singh A, Winterbottom E, Daar IO. 2012. Eph/ephrin signaling in cell-cell and cell-substrate adhesion. Front Biosci (Landmark Ed) 17:473-497.

Wolf MY, Wolf YI, Koonin EV. 2008. Comparable contributions of structural-functional constraints and expression level to the rate of protein sequence evolution. Biol Direct 3:40.

ST1 – Species used in this study

Species	Species Unipro	Organism ID	Taxon identi	Taxonomy	Number of proteins	DB	Download da	Status	
Homo sapiens	HUMAN	Hsa	9606	Metazoa › Chordata › Craniata › Vertebrata › Euteleostomi › Mammalia › Eutheria › Euarchontoglires › Primates › Haplorrhini ›	42163	UniProt	Feb. 2017	Reference proteome set	
Mus musculus	MOUSE	Mus	10090	Metazoa › Chordata › Craniata › Vertebrata › Euteleostomi › Mammalia › Eutheria › Euarchontoglires › Glires › Rodentia › Sciur	25469	UniProt	Feb. 2017	Reference proteome set	
Monodelphis domestica	MONDO	Mdo	13616	Metazoa › Chordata › Craniata › Vertebrata › Euteleostomi › Mammalia › Metatheria › Didelphimorphia › Didelphidae › Monod	22243	UniProt	Feb. 2017	Reference proteome set	
Ornithorhynchus anatinus	ORNAN	Oan	9258	Metazoa › Chordata › Craniata › Vertebrata › Euteleostomi › Mammalia › Monotremata › Ornithorhynchidae › Ornithorhynchus	23552	UniProt	Feb. 2017	Reference proteome set	
Gallus gallus	CHICK	Gga	9031	Metazoa › Chordata › Craniata › Vertebrata › Euteleostomi › Testudines + Archosauria group › Archosauria › Dinosauria › Sauris	26068	UniProt	Feb. 2017	Reference proteome set	
Taeniopygia guttata	TAEGU	Tgu	59729	Metazoa › Chordata › Craniata › Vertebrata › Euteleostomi › Testudines + Archosauria group › Archosauria › Dinosauria › Sauris	18141	UniProt	Feb. 2017	Reference proteome set	
Alligator mississippiensis	ALLMI	Ami	8496	Metazoa › Chordata › Craniata › Vertebrata › Euteleostomi › Testudines + Archosauria group › Archosauria › Crocodylia › Alligat	31974	UniProt	Feb. 2017	Reference proteome set	
Chelonia mydas	CHEMY	Cmy	8469	Metazoa › Chordata › Craniata › Vertebrata › Euteleostomi › Testudines + Archosauria group › Testudines › Cryptodira › Cheloni	18960	UniProt	Feb. 2017	Reference proteome set	
Pelodiscus sinensis	PELSI	Psi	13735	Metazoa › Chordata › Craniata › Vertebrata › Euteleostomi › Testudines + Archosauria group › Testudines › Cryptodira › Trionycl	20509	UniProt	Feb. 2017	Reference proteome set	
Anolis carolinensis	ANOCA	Aca	28377	Metazoa › Chordata › Craniata › Vertebrata › Euteleostomi › Lepidosauria › Squamata › Bifurcata › Unidentata › Episquamata ›	19108	UniProt	Feb. 2017	Reference proteome set	
Ophiophagus hannah	OPHHA	Oha	8665	Metazoa › Chordata › Craniata › Vertebrata › Euteleostomi › Lepidosauria › Squamata › Bifurcata › Unidentata › Episquamata ›	18387	UniProt	Feb. 2017	Reference proteome set	
Xenopus tropicalis	XENTR	Xtr	8364	Metazoa › Chordata › Craniata › Vertebrata › Euteleostomi › Amphibia › Batrachia › Anura › Pipoidea › Pipidae › Xenopodinae ›	29625	UniProt	Feb. 2017	Reference proteome set	
Latimeria chalumnae	LATCH	Lch	7897	Metazoa › Chordata › Craniata › Vertebrata › Euteleostomi › Coelacanthiformes › Coelacanthidae › Latimeria	23429	UniProt	Feb. 2017	Reference proteome set	
Danio rerio	DANRE	Dre	7955	Metazoa › Chordata › Craniata › Vertebrata › Euteleostomi › Actinopterygii › Neopterygii › Teleostei › Ostariophysi › Cypriniforn	43418	UniProt	Feb. 2017	Reference proteome set	
Oryzias latipes	ORYLA	Ola	8090	Metazoa › Chordata › Craniata › Vertebrata › Euteleostomi › Actinopterygii › Neopterygii › Teleostei › Euteleostei › Neoteleoste	24636	UniProt	Feb. 2017	Reference proteome set	
Lepisosteus oculatus	LEPOC	Loc	7918	Metazoa › Chordata › Craniata › Vertebrata › Euteleostomi › Actinopterygii › Neopterygii › Semionotiformes › Lepisosteidae › L	22463	UniProt	Feb. 2017	Complete proteome set	
Callorhinchus milii	CALMI	Cmi	7868	Metazoa › Chordata › Craniata › Vertebrata › Chondrichthyes › Holocephali › Chimaeriformes › Callorhinchidae › Callorhinchus	20019	UniProt	Feb. 2017	Draft	
Petromyzon marinus	PETMA	Pma	7757	Metazoa › Chordata › Craniata › Vertebrata › Cyclostomata › Hyperoartia › Petromyzontiformes › Petromyzontidae › Petromyzo	13495	UniProt	Feb. 2017	Draft	
Ciona intestinalis	CIOIN	Cin	7719	Metazoa › Chordata › Tunicata › Ascidiacea › Enterogona › Phlebobranchia › Cionidae › Ciona	17309	UniProt	Feb. 2017	Reference proteome set	
Oikopleura dioica	OIKDI	Odi	34765	Metazoa › Chordata › Tunicata › Appendicularia › Oikopleuridae › Oikopleura	17050	UniProt	Feb. 2017	Complete proteome set	
Branchiostoma floridae	BRAFL	Bfl	7739	Metazoa › Chordata › Cephalochordata › Branchiostomidae › Branchiostoma	28544	UniProt	Feb. 2017	Reference proteome set	
Saccoglossus kowalevskii	SACKO	Sko	10224	Metazoa › Hemichordata › Enteropneusta › Harrimaniidae › Saccoglossus	22113	NCBI RefSeq	Feb. 2017	Scaffolds	
Strongylocentrotus purpuratus	STRPU	Spu	7668	Metazoa › Echinodermata › Eleutherozoa › Echinozoa › Echinoidea › Euechinoidea › Echinacea › Echinoida › Strongylocentrotida	28593	UniProt	Feb. 2017	Reference proteome set	
Caenorhabditis elegans	CAEEL	Cel	6239	Metazoa › Ecdysozoa › Nematoda › Chromadorea › Rhabditida › Rhabditoidea › Rhabditidae › Peloderinae › Caenorhabditis	26725	UniProt	Feb. 2017	Reference proteome set	
Trichinella spiralis	TRISP	Tsp	6334	Metazoa › Ecdysozoa › Nematoda › Enoplea › Dorylaimia › Trichocephalida › Trichinellidae › Trichinella	16041	UniProt	Feb. 2017	Reference proteome set	
Drosophila melanogaster	DROME	Dme	7227	Metazoa › Ecdysozoa › Arthropoda › Hexapoda › Insecta › Pterygota › Neoptera › Endopterygota › Diptera › Brachycera › Muscor	22005	UniProt	Feb. 2017	Reference proteome set	
Apis mellifera	APIME	Ame	7461	Metazoa › Ecdysozoa › Arthropoda › Hexapoda › Insecta › Pterygota › Neoptera › Endopterygota › Hymenoptera › Apocrita › Acu	15323	UniProt	Feb. 2017	Reference proteome set	
Tribolium castaneum	TRICA	Tca	7070	Metazoa › Ecdysozoa › Arthropoda › Hexapoda › Insecta › Pterygota › Neoptera › Endopterygota › Coleoptera › Polyphaga › Cucu	18505	UniProt	Feb. 2017	Reference proteome set	
Ixodes scapularis	IXOSC	Isc	6945	Metazoa › Ecdysozoa › Arthropoda › Chelicerata › Arachnida › Acari › Parasitiformes › Ixodida › Ixodoidea › Ixodidae › Ixodinae ›	20473	UniProt	Feb. 2017	Reference proteome set	
Daphnia pulex	DAPPU	Dpu	6669	Metazoa › Ecdysozoa › Arthropoda › Crustacea › Branchiopoda › Diplostraca › Cladocera › Anomopoda › Daphniidae › Daphnia	30137	UniProt	Feb. 2017	Reference proteome set	
Strigamia maritima	STRMM	Sma	126957	Metazoa › Ecdysozoa › Arthropoda › Myriapoda › Chilopoda › Pleurostigmophora › Geophilomorpha › Linotaeniidae › Strigamia	14972	UniProt	Feb. 2017	Reference proteome set	
Helobdella robusta	HELRO	Hro	6412	Metazoa › Lophotrochozoa › Annelida › Clitellata › Hirudinida › Hirudinea › Rhynchobdellida › Glossiphoniidae › Helobdella	23328	UniProt	Feb. 2017	Reference proteome set	
Capitella teleta	CAPTE	Cte	283909	Metazoa › Lophotrochozoa › Annelida › Polychaeta › Scolecida › Capitellida › Capitellidae › Capitella	32109	UniProt	Feb. 2017	Draft	
Lottia gigantea	LOTGI	Lgi	225164	Metazoa › Lophotrochozoa › Mollusca › Gastropoda › Patellogastropoda › Lottioidea › Lottiidae › Lottia	23675	UniProt	Feb. 2017	Reference proteome set	
Crassostrea gigas	CRAGI	Cgi	29159	Metazoa › Lophotrochozoa › Mollusca › Bivalvia › Pteriomorphia › Ostreoida › Ostreoidea › Ostreidae › Crassostrea	25982	UniProt	Feb. 2017	Reference proteome set	
Schistosoma mansoni	SCHMA	Smn	212216	Metazoa › Platyhelminthes › Trematoda › Digenea › Strigeidida › Schistosomatoidea › Schistosomatidae › Schistosoma	11723	UniProt	Feb. 2017	Reference proteome set	
Hymenolepis microstoma	HYMMI	Hmi	85433	Metazoa › Platyhelminthes › Cestoda › Eucestoda › Cyclophyllidea › Hymenolepididae › Hymenolepis	12000	UniProt	Feb. 2017	Reference proteome set	
Nematostella vectensis	NEMVE	Nve	45351	Metazoa › Cnidaria › Anthozoa › Hexacorallia › Actiniaria › Edwardsiidae › Nematostella	24435	UniProt	Feb. 2017	Reference proteome set	
Hydra vulgaris (Hydra) (Hydra attenuat	HYDVU	Hvu	6087	Metazoa › Cnidaria › Hydrozoa › Hydroidolina › Anthoathecata › Aplanulata › Hydridae › Hydra	22051	NCBI RefSeq	Feb. 2017	Scaffolds	
Mnemiopsis leidyi	MNELE	Mle	27924	Metazoa › Ctenophora › Cyclocoela › Lobata › Bolinopsidae › Mnemiopsis	16548	NHGRI	Feb. 2017	Scaffolds	http://research.nhgri.nih.gov/mnemiopsis/download/download.cgi?dl=proteome
Trichoplax adhaerens	TRIAD	Tad	10228	Metazoa › Placozoa › Trichoplax	11520	UniProt	Feb. 2017	Reference proteome set	
Amphimedon queenslandica	AMPQE	Aqu	400682	Metazoa › Porifera › Demospongiae › Ceractinomorpha › Haplosclerida › Niphatidae › Amphimedon	29758	UniProt	Feb. 2017	Reference proteome set	
Oscarella carmela	OSCCA	Oca	386100	Metazoa › Porifera › Homoscleromorpha › Homosclerophorida › Plakinidae › Oscarella	29220	Compagen	Feb. 2017	Scaffolds	http://www.compagen.org
Sycon ciliatum	SYCCI	Sci	27933	Metazoa › Porifera › Calcarea › Calcaronea › Leucosolenida › Sycettidae › Sycon	50722	Compagen	Feb. 2017	Scaffolds	
Monosiga brevicollis	MONBE	Mbr	81824	Choanoflagellida › Codonosigidae › Monosiga	9188	UniProt	Feb. 2017	Reference proteome set	
Salpingoeca rosetta (strain ATCC 5081ε	SALR5	Sro	946362	Choanoflagellida › Salpingoecidae › Salpingoeca	11698	UniProt	Feb. 2017	Reference proteome set	
Capsaspora owczarzaki ATCC 30864	CAPO3	Cow	595528	Filasterea › Capsaspora	9794	UniProt	Feb. 2017	Reference proteome set	
Sphaeroforma arctica JP610	SPHAR	Sar	667725	Ichthyosporea › Ichthyophonida › Sphaeroforma	18649	UniProt	Feb. 2017	Reference proteome set	
Saccharomyces cerevisiae (strain ATCC	YEAST	Sce	559292	Fungi › Dikarya › Ascomycota › Saccharomycotina › Saccharomycetes › Saccharomycetales › Saccharomycetaceae › Saccharomy	6724	UniProt	Feb. 2017	Reference proteome set	
Schizosaccharomyces pombe (strain 97.	SCHPO	Spo	284812	Fungi › Dikarya › Ascomycota › Taphrinomycotina › Schizosaccharomycetes › Schizosaccharomycetales › Schizosaccharomyceta	5142	UniProt	Feb. 2017	Reference proteome set	
Cryptococcus neoformans var. neoform	CRYNJ	Cne	214684	Fungi › Dikarya › Basidiomycota › Agaricomycotina › Tremellomycetes › Tremellales › Tremellaceae › Filobasidiella › Filobasidi	6743	UniProt	Feb. 2017	Reference proteome set	
Encephalitozoon cuniculi (strain GB-M1	ENCCU	Ecu	284813	Fungi › Microsporidia › Unikaryonidae › Encephalitozoon	2008	UniProt	Feb. 2017	Reference proteome set	
Batrachochytrium dendrobatidis (strain	BATDJ	Bde	684364	Fungi › Chytridiomycota › Chytridiomycetes › Rhizophydiales › Rhizophydiales incertae sedis › Batrachochytrium	8610	UniProt	Feb. 2017	Reference proteome set	
Rhizophagus irregularis (strain DAOM 1	RHIID	Rir	747089	Fungi › Glomeromycota › Glomeromycetes › Glomerales › Glomeraceae › Rhizophagus	29846	UniProt	Feb. 2017	Reference proteome set	
Fonticula alba	9EUKA	Fal	691883	Nucleariidae and Fonticula group › Fonticula	6244	UniProt	Feb. 2017	Scaffolds	
Nutomonas longa	NUTLO	Nlo	1246795	Apusozoa › Nutomonas	24493	Multicellgenome	Feb. 2017	Scaffolds	http://multicellgenome.com/resources/genomic-resources
Dictyostelium discoideum	DICDI	Ddi	44689	Amoebozoa › Mycetozoa › Dictyosteliida › Dictyostelium	12745	UniProt	Feb. 2017	Reference proteome set	
Entamoeba histolytica	ENTHI	Ehi	5759	Amoebozoa › Archamoebae › Entamoebidae › Entamoeba	7959	UniProt	Feb. 2017	Reference proteome set	
Arabidopsis thaliana	ARATH	Ath	3702	Viridiplantae › Streptophyta › Embryophyta › Tracheophyta › Spermatophyta › Magnoliophyta › eudicotyledons › core eudicotyle	31387	UniProt	Feb. 2017	Reference proteome set	
Physcomitrella patens subsp. patens	PHYPA	Ppa	3218	Viridiplantae › Streptophyta › Embryophyta › Bryophyta › Bryophytina › Bryopsida › Funariidae › Funariales › Funariaceae › Phys	34837	UniProt	Feb. 2017	Reference proteome set	
Chlamydomonas reinhardtii	CHLRE	Cre	3055	Viridiplantae › Chlorophyta › Chlorophyceae › Chlamydomonadales › Chlamydomonadaceae › Chlamydomonas	14337	UniProt	Feb. 2017	Reference proteome set	
Micromonas pusilla (strain CCMP1545)	MICPC	Mpu	564608	Viridiplantae › Chlorophyta › Mamiellophyceae › Mamiellales › Micromonas	10250	UniProt	Feb. 2017	Complete proteome set	
Galdieria sulphuraria	GALSU	Gsu	130081	Rhodophyta › Bangiophyceae › Cyanidiales › Cyanidiaceae › Galdieria	7035	UniProt	Feb. 2017	Reference proteome set	
Guillardia theta CCMP2712	GUITH	Gth	905079	Cryptophyta › Pyrenomonadales › Geminigeraceae › Guillardia	24590	NCBI RefSeq	Feb. 2017	Reference proteome set	
Cyanophora paradoxa	CYAPA	Cpa	2762	Glaucocystophyceae › Cyanophoraceae › Cyanophora	476	UniProt	Feb. 2017	Scaffolds	
Emiliania huxleyi	EMIHU	Ehu	2903	Haptophyceae › Isochrysidales › Noelaerhabdaceae › Emiliania	35700	UniProt	Feb. 2017	Reference proteome set	
Aureococcus anophagefferens	AURAN	Ano	44056	Stramenopiles › Pelagophyceae › Pelagomonadales › Aureococcus	11501	UniProt	Feb. 2017	Draft	
Phaeodactylum tricornutum (strain CCA	PHATC	Ptr	556484	Stramenopiles › Bacillariophyta › Bacillariophyceae › Bacillariophycidae › Naviculales › Phaeodactylaceae › Phaeodactylum	10465	UniProt	Feb. 2017	Reference proteome set	
Ectocarpus siliculosus	ECTSI	Esi	2880	Stramenopiles › PX clade › Phaeophyceae › Ectocarpales › Ectocarpaceae › Ectocarpus	16334	UniProt	Feb. 2017	Reference proteome set	
Plasmodium falciparum (isolate 3D7)	PLAF7	Pfa	36329	Alveolata › Apicomplexa › Aconoidasida › Haemosporida › Plasmodium › Plasmodium (Laverania)	5369	UniProt	Feb. 2017	Reference proteome set	
Ichthyophthirius multifiliis (strain G5)	ICHMG	Imu	857967	Alveolata › Ciliophora › Intramacronucleata › Oligohymenophorea › Hymenostomatida › Ophryoglenina › Ichthyophthirius	8049	UniProt	Feb. 2017	Reference proteome set	
Bigelowiella natans (Pedinomonas min	BIGNA	Bna	227086	Rhizaria › Cercozoa › Chlorarachniophyceae › Bigelowiella	575	UniProt	Feb. 2017	Complete proteome set	
Trichomonas vaginalis	TRIVA	Tva	5722	Parabasalia › Trichomonadida › Trichomonadidae › Trichomonas	46581	UniProt	Feb. 2017	Scaffolds	
Naegleria gruberi	NAEGR	Ngr	5762	Heterolobosea › Schizopyrenida › Vahlkampfiidae › Naegleria	15636	UniProt	Feb. 2017	Scaffolds	

Trypanosoma cruzi Dm28c	TRYCR	Tcr	5693	Euglenozoa › Kinetoplastida › Trypanosomatidae › Trypanosoma › Schizotrypanum	11346	UniProt	Feb. 2017	Reference proteome set
Giardia intestinalis	GIAIN	Gin	5741	Diplomonadida › Hexamitidae › Giardiinae › Giardia	6085	UniProt	Feb. 2017	Complete proteome set
Lokiarchaeum sp. GC14_75	Lok	Lok	1538547	Archaea › Lokiarchaeota	5378	UniProt	Feb. 2017	Scaffolds
Pyrolobus fumarii 1A	PYRF1	Pfu	694429	Archaea › Crenarchaeota › Thermoprotei › Desulfurococcales › Pyrodictiaceae › Pyrolobus	1967	UniProt	Feb. 2017	Complete proteome set
Sulfolobus solfataricus P2	SULSO	Sso	273057	Archaea › Crenarchaeota › Thermoprotei › Sulfolobales › Sulfolobaceae › Sulfolobus	2938	UniProt	Feb. 2017	Complete proteome set
Haloferax mediterranei ATCC 33500	HALMT	Hme	523841	Archaea › Euryarchaeota › Halobacteria › Haloferacales › Haloferacaceae › Haloferax	3826	UniProt	Feb. 2017	Complete proteome set
Methanothermobacter thermautotroph	METTH	Mth	187420	Archaea › Euryarchaeota › Methanobacteria › Methanobacteriales › Methanobacteriaceae › Methanothermobacter	1868	UniProt	Feb. 2017	Complete proteome set
Methanosarcina acetivorans C2A	METAC	Mac	188937	Archaea › Euryarchaeota › Methanomicrobia › Methanosarcinales › Methanosarcinaceae › Methanosarcina	4468	UniProt	Feb. 2017	Complete proteome set
Korarchaeum cryptofilum OPF8	KORCO	Kcr	374847	Archaea › Korarchaeota › Candidatus Korarchaeum	1602	UniProt	Feb. 2017	Complete proteome set
Nanoarchaeum equitans Kin4-M	NANEQ	Neq	228908	Archaea › Nanoarchaeota › Nanoarchaeales › Nanoarchaeaceae › Nanoarchaeum	536	UniProt	Feb. 2017	Complete proteome set
Cenarchaeum symbiosum A	CENSY	Csy	414004	Archaea › Thaumarchaeota › Cenarchaeales › Cenarchaeaceae › Cenarchaeum	2022	UniProt	Feb. 2017	Complete proteome set
Nitrosopumilus maritimus SCM1	NITMS	Nma	436308	Archaea › Thaumarchaeota › Nitrosopumilales › Nitrosopumilaceae › Nitrosopumilus	1795	UniProt	Feb. 2017	Complete proteome set
Granulicella mallensis MP5ACTX8	GRAMM	Gma	682795	Bacteria › Acidobacteria › Acidobacteriales › Acidobacteriaceae › Granulicella	4804	UniProt	Feb. 2017	Complete proteome set
Gardnerella vaginalis ATCC 14019	GARV3	Gva	525284	Bacteria › Actinobacteria › Bifidobacteriales › Bifidobacteriaceae › Gardnerella	1365	UniProt	Feb. 2017	Complete proteome set
Frankia alni ACN14a	FRAAA	Fra	326424	Bacteria › Actinobacteria › Frankiales › Frankiaceae › Frankia	6710	UniProt	Feb. 2017	Complete proteome set
Streptomyces coelicolor A3(2)	STRCO	Sco	100226	Bacteria › Actinobacteria › Streptomycetales › Streptomycetaceae › Streptomyces › Streptomyces albidoflavus group	8038	UniProt	Feb. 2017	Complete proteome set
Persephonella marina EX-H1	PERMH	Pma	123214	Bacteria › Aquificae › Aquificales › Hydrogenothermaceae › Persephonella	2048	UniProt	Feb. 2017	Complete proteome set
Chthonomonas calidirosea T49	CHTCT	Cca	1303518	Bacteria › Armatimonadetes › Chthonomonadetes › Chthonomonadales › Chthonomonadaceae › Chthonomonas	2809	UniProt	Feb. 2017	Complete proteome set
Aequorivita sublithincola DSM 14238	AEQSU	Asu	746697	Bacteria › Bacteroidetes › Flavobacteriia › Flavobacteriales › Flavobacteriaceae › Aequorivita	3134	UniProt	Feb. 2017	Complete proteome set
Caldisericum exile DSM 21853	CALEA	Cex	511051	Bacteria › Caldiserica › Caldisericia › Caldisericales › Caldisericaceae › Caldisericum	1566	UniProt	Feb. 2017	Complete proteome set
Chlamydia pneumoniae CWL029	CHLPP	Cpn	83558	Bacteria › Chlamydiae › Chlamydiales › Chlamydiaceae › Chlamydia/Chlamydophila group › Chlamydia	1052	UniProt	Feb. 2017	Complete proteome set
Simkania negevensis ATCC VR-1471	SIMNZ	Sne	331113	Bacteria › Chlamydiae › Parachlamydiales › Simkaniaceae › Simkania	2516	UniProt	Feb. 2017	Complete proteome set
Chloroflexus aurantiacus ATCC 29366	CHLAA	Cau	324602	Bacteria › Chloroflexi › Chloroflexia › Chloroflexales › Chloroflexineae › Chloroflexaceae › Chloroflexus	3850	UniProt	Feb. 2017	Complete proteome set
Desulfurispirillum indicum S5	DESIS	Din	653733	Bacteria › Chrysiogenetes › Chrysiogenales › Chrysiogenaceae › Desulfurispirillum	2551	UniProt	Feb. 2017	Complete proteome set
Anabaena variabilis ATCC 29413	ANAVT	Ava	240292	Bacteria › Cyanobacteria › Nostocales › Nostocaceae › Anabaena	5634	UniProt	Feb. 2017	Complete proteome set
Calothrix sp. PCC 6303	9CYAN	Cal	1170562	Bacteria › Cyanobacteria › Nostocales › Rivulariaceae › Calothrix	5484	UniProt	Feb. 2017	Complete proteome set
Deferribacter desulfuricans SSM1	DEFDS	Dde	639282	Bacteria › Deferribacteres › Deferribacterales › Deferribacteraceae › Deferribacter	2338	UniProt	Feb. 2017	Complete proteome set
Deinococcus radiodurans R1	DEIRA	Dra	243230	Bacteria › Deinococcus-Thermus › Deinococci › Deinococcales › Deinococcaceae › Deinococcus	3085	UniProt	Feb. 2017	Complete proteome set
Dictyoglomus turgidum DSM 6724	DICTD	Dtu	515635	Bacteria › Dictyoglomi › Dictyoglomales › Dictyoglomaceae › Dictyoglomus	1743	UniProt	Feb. 2017	Complete proteome set
Elusimicrobium minutum Pei191	ELUMP	Emi	445932	Bacteria › Elusimicrobia › Elusimicrobia › Elusimicrobiales › Elusimicrobiaceae › Elusimicrobium	1528	UniProt	Feb. 2017	Complete proteome set
Bacillus subtilis (strain 168)	BACSU	Bsu	224308	Bacteria › Firmicutes › Bacilli › Bacillales › Bacillaceae › Bacillus	4197	UniProt	Feb. 2017	Complete proteome set
Clostridium botulinum Type A	CLOBO	Cbo	441771	Bacteria › Firmicutes › Clostridia › Clostridiales › Clostridiaceae › Clostridium	3590	UniProt	Feb. 2017	Complete proteome set
Fusobacterium nucleatum subsp. nucle	FUSNN	Fnu	190304	Bacteria › Fusobacteria › Fusobacteriales › Fusobacteriaceae › Fusobacterium	2046	UniProt	Feb. 2017	Complete proteome set
Gemmatimonas aurantiaca T-27	GEMAT	Gau	379066	Bacteria › Gemmatimonadetes › Gemmatimonadales › Gemmatimonadaceae › Gemmatimonas	3932	UniProt	Feb. 2017	Complete proteome set
Thermodesulfovibrio yellowstonii ATCC	THEYD	Tye	289376	Bacteria › Nitrospirae › Nitrospirales › Nitrospiraceae › Thermodesulfovibrio	1982	UniProt	Feb. 2017	Complete proteome set
Phycisphaera mikurensis NBRC 102666	PHYMF	Pmi	1142394	Bacteria › Planctomycetes › Phycisphaerae › Phycisphaerales › Phycisphaeraceae › Phycisphaera	3269	UniProt	Feb. 2017	Complete proteome set
Isosphaera pallida ATCC 43644	ISOPI	Ipa	575540	Bacteria › Planctomycetes › Planctomycetia › Planctomycetales › Isosphaeraceae › Isosphaera	3721	UniProt	Feb. 2017	Complete proteome set
Singulisphaera acidiphila DSM 18658	SINAD	Sac	886293	Bacteria › Planctomycetes › Planctomycetia › Planctomycetales › Isosphaeraceae › Singulisphaera	7126	UniProt	Feb. 2017	Complete proteome set
Gemmata obscuriglobus UQM 2246	9PLAN	Gob	214688	Bacteria › Planctomycetes › Planctomycetia › Planctomycetales › Planctomycetaceae › Gemmata	6832	NCBI RefSeq	Feb. 2017	Scaffolds
Pirellula staleyi DSM 6068	PIRSD	Pst	530564	Bacteria › Planctomycetes › Planctomycetia › Planctomycetales › Planctomycetaceae › Pirellula	4711	UniProt	Feb. 2017	Complete proteome set
Planctomyces limnophilus DSM 3776	PLAL2	Pli	521674	Bacteria › Planctomycetes › Planctomycetia › Planctomycetales › Planctomycetaceae › Planctopirus	4258	UniProt	Feb. 2017	Complete proteome set
Rhodopirellula baltica SH 1	RHOBT	Rba	243090	Bacteria › Planctomycetes › Planctomycetia › Planctomycetales › Planctomycetaceae › Rhodopirellula	7271	UniProt	Feb. 2017	Complete proteome set
Caulobacter crescentus CB15	CAUCR	Ccr	190650	Bacteria › Proteobacteria › Alphaproteobacteria › Caulobacterales › Caulobacteraceae › Caulobacter	3720	UniProt	Feb. 2017	Complete proteome set
Rhizobium tropici CIAT 899	RHITR	Rtr	698761	Bacteria › Proteobacteria › Alphaproteobacteria › Rhizobiales › Rhizobiaceae › Rhizobium/Agrobacterium group › Rhizobium	6230	UniProt	Feb. 2017	Complete proteome set
Neisseria meningitidis serogroup B MC	NEIMB	Nme	122586	Bacteria › Proteobacteria › Betaproteobacteria › Neisseriales › Neisseriaceae › Neisseria	2001	UniProt	Feb. 2017	Complete proteome set
Myxococcus xanthus DK 1622	MYXXD	Mxa	246197	Bacteria › Proteobacteria › Deltaproteobacteria › Myxococcales › Cystobacterineae › Myxococcaceae › Myxococcus	7314	UniProt	Feb. 2017	Complete proteome set
Escherichia coli K12	ECOLI	Eco	316385	Bacteria › Proteobacteria › Gammaproteobacteria › Enterobacteriales › Enterobacteriaceae › Escherichia	4306	UniProt	Feb. 2017	Complete proteome set
Haemophilus influenzae ATCC 51907	HAEIN	Hin	71421	Bacteria › Proteobacteria › Gammaproteobacteria › Pasteurellales › Pasteurellaceae › Haemophilus	1707	UniProt	Feb. 2017	Complete proteome set
Pseudomonas putida GB-1	PSEPG	Ppu	76869	Bacteria › Proteobacteria › Gammaproteobacteria › Pseudomonadales › Pseudomonadaceae › Pseudomonas	5396	UniProt	Feb. 2017	Complete proteome set
Spirochaeta thermophila ATCC 49972	SPITD	Sth	665571	Bacteria › Spirochaetes › Spirochaetales › Spirochaetaceae › Spirochaeta	2199	UniProt	Feb. 2017	Complete proteome set
Aminobacterium colombiense DSM 12:	AMICL	Aco	572547	Bacteria › Synergistetes › Synergistia › Synergistales › Synergistaceae › Aminobacterium	1872	UniProt	Feb. 2017	Complete proteome set
Mycoplasma genitalium G37	MYCGE	Mge	243273	Bacteria › Tenericutes › Mollicutes › Mycoplasmataceae › Mycoplasma	483	UniProt	Feb. 2017	Reference proteome set
Thermodesulfobacterium geofontis OP	THEGP	Tge	795359	Bacteria › Thermodesulfobacteria › Thermodesulfobacteriales › Thermodesulfobacteriaceae › Thermodesulfobacterium	1594	UniProt	Feb. 2017	Complete proteome set
Marinitoga piezophila KA3	MARPK	Mpi	443254	Bacteria › Thermotogae › Petrotogales › Petrotogaceae › Marinitoga	2044	UniProt	Feb. 2017	Complete proteome set
Methylacidiphilum infernorum V4	METI4	Min	481448	Bacteria › Verrucomicrobia › Methylacidiphilae › Methylacidiphilales › Methylacidiphilaceae › Methylacidiphilum	2470	UniProt	Feb. 2017	Complete proteome set

ST2 – Eph Sequence identifiers

Tree seq ID	DB Seq ID	DB
ALLMI_A1	XP_006268664	RefSeq
ALLMI_A2	XP_006273097	RefSeq
ALLMI_A3	XP_006266352	RefSeq
ALLMI_A4	XP_006266130	RefSeq
ALLMI_A5	XP_019355652	RefSeq
ALLMI_A6	XP_019332111	RefSeq
ALLMI_A7	XP_006263431	RefSeq
ALLMI_A8	XP_019350837	RefSeq
ALLMI_10	A0A151NFP2	UniProt
ALLMI_B1	A0A151NGY0	UniProt
ALLMI_B2	XP_014452676	RefSeq
ALLMI_B3	XP_006260832	RefSeq
ALLMI_B4	XP_006262973	RefSeq
ALLMI_B5	XP_006271852	RefSeq
AMPQE_A7	I1EC65/A0A1X7SP49	UniProt
AMPQE_B1	I1GD99/A0A1X7VPX9	UniProt
AMPQE_X	I1ERJ1/A0A1X7TBG3	UniProt
ANOCA_A1	H9GCN5	UniProt
ANOCA_A2	H9GBA4	UniProt
ANOCA_A3	G1K9V3	UniProt
ANOCA_A4	G1KDW5	UniProt
ANOCA_A5	H9GJH9	UniProt
ANOCA_A6	G1KII9	UniProt
ANOCA_A7	G1KC20	UniProt
ANOCA_A8	H9GLN7	UniProt
ANOCA_B1	G1KIZ8	UniProt
ANOCA_B2	XP_008123105	RefSeq
ANOCA_B3	H9GDK1	UniProt
ANOCA_B4	XP_003230237	RefSeq
ANOCA_B5	XP_003227626	RefSeq
APIME_A5	A0A087ZS47	UniProt
BRAFL_1	C3XPM2	UniProt
BRAFL_2	C3XPM1	UniProt
BRAFL_A7	C3XPM3	UniProt
CAEEL_A4	O61460	UniProt
CALMI_A2	V9K8H6	UniProt
CALMI_A3	XP_007893894	RefSeq
CALMI_A4	XP_007886441	RefSeq
CALMI_A5	XP_007897160	RefSeq
CALMI_A6	XP_007899447	RefSeq
CALMI_A7	XP_007891226	RefSeq
CALMI_A8	XP_007905434	RefSeq
CALMI_A10	XP_007893147	RefSeq
CALMI_B1	XP_007908799	RefSeq
CALMI_B2	XP_007905430	RefSeq
CALMI_B3	XP_007900624	RefSeq
CAPTE_B2	R7UCY0	UniProt
CHEMY_A1	M7B241	UniProt
CHEMY_A2	M7CBJ3	UniProt
CHEMY_A3	M7BQ91	UniProt
CHEMY_A5	M7B832	UniProt
CHEMY_A6	XP_007057612	RefSeq
CHEMY_A7	M7BAB2	UniProt
CHEMY_A8	M7CBL6	UniProt
CHEMY_10	M7CMI4	UniProt
CHEMY_B1	M7BRR4	UniProt
CHEMY_B2	XP_007053185	RefSeq
CHEMY_B3	M7C9S8	UniProt
CHEMY_B6	M7AWU1	UniProt
CHICK_A1	F1NB88	UniProt
CHICK_A2	A0A1D5PXE5	UniProt
CHICK_A3	F1NJX6	UniProt
CHICK_A4	Q07496	UniProt
CHICK_A5	P54755	UniProt
CHICK_A6	XP_416644	RefSeq
CHICK_A7	O42422	UniProt
CHICK_A8	F1NHC6	UniProt
CHICK_10	XP_425778	RefSeq
CHICK_B1	F1P4I4	UniProt
CHICK_B2	F1NHC7	UniProt
CHICK_B3	F1NIS8	UniProt
CHICK_B4	XP_015129488	RefSeq
CHICK_B5	Q07497	UniProt
CHICK_B6	F1NAX7	UniProt
CIOIN_A4	F6QR95	UniProt
CIOIN_A7	F6ZVC5	UniProt
CIOIN_AX	F6YLC8	UniProt
CIOIN_B3	F6VYU2	UniProt
CIOIN_X1	F6ZB89	UniProt
CIOIN_X2	F6QR87	UniProt
CRAGI_A4	K1QIZ1	UniProt
DANRE_A2A	F1QSD6	UniProt
DANRE_A2B	E7F533	UniProt
DANRE_A3	F1Q9D9	UniProt
DANRE_A4A	F8W481	UniProt
DANRE_A4B	F1RCL1	UniProt
DANRE_A4L	O13146	UniProt
DANRE_A5	XP_001338064	RefSeq
DANRE_A6	F1REM5	UniProt
DANRE_A7	A0A0R4IEC7	UniProt
DANRE_A8	F1Q4S7	UniProt
DANRE_10	E7F153	UniProt
DANRE_B1	XP_017207747	RefSeq
DANRE_B2A	A2CEJ3	UniProt
DANRE_B2B	F1RCR4	UniProt

DANRE_B3A	B8JKB8	UniProt
DANRE_B3B	A0A0G2L6L8	UniProt
DANRE_B4A	A0A0R4IJK3	UniProt
DANRE_B4B	F1QR66	UniProt
DANRE_B5	F1QU92	UniProt
DAPPU_A4	E9GM17	UniProt
DROME_B2	Q0KIF4	UniProt
HELRO_B2	T1EGM5	UniProt
HUMAN_A1	P21709	UniProt
HUMAN_A2	P29317	UniProt
HUMAN_A3	P29320	UniProt
HUMAN_A4	P54764	UniProt
HUMAN_A5	P54756	UniProt
HUMAN_A6	Q9UF33	UniProt
HUMAN_A7	Q15375	UniProt
HUMAN_A8	P29322	UniProt
HUMAN_10	Q5JZY3	UniProt
HUMAN_B1	P54762	UniProt
HUMAN_B2	P29323	UniProt
HUMAN_B3	P54753	UniProt
HUMAN_B4	P54760	UniProt
HUMAN_B6	O15197	UniProt
HYDVU_1	NP_001296657	RefSeq
HYDVU_2	NP_001296646	RefSeq
HYDVU_3	NP_001296717	RefSeq
HYDVU_4	NP_001296582	RefSeq
HYDVU_5	S4VFR6	UniProt
HYMMI_B6	A0A068XJ74	UniProt
IXOSC_A5	B7QJ81	UniProt
LATCH_A1	H3AYV3	UniProt
LATCH_A2	H3AUH0	UniProt
LATCH_A3	XP_014350287	RefSeq
LATCH_A4	H3AD29	UniProt
LATCH_A5	H3AY04	UniProt
LATCH_A6	XP_006001862	RefSeq
LATCH_A7	H3ADN2	UniProt
LATCH_A8	H3A0R0+H3AU43	UniProt
LATCH_10	H3A1I8	UniProt
LATCH_B1	XP_006005454+XP_014344559	RefSeq
LATCH_B2	XP_014349188+XP_005988773	RefSeq
LATCH_B3	H3B223	UniProt
LATCH_B4	H3A109	UniProt
LATCH_B5	H3A9X5	UniProt
LEPOC_A2	W5MMU5	UniProt
LEPOC_A3	W5MWV0	UniProt
LEPOC_A4	W5M9H5	UniProt
LEPOC_A5	W5M460	UniProt
LEPOC_A6	W5MWQ2	UniProt
LEPOC_A7	W5NK05	UniProt
LEPOC_A8	W5LXN4	UniProt
LEPOC_A10	W5MFW3	UniProt
LEPOC_B1	W5MEV9	UniProt
LEPOC_B2	W5M6I4	UniProt
LEPOC_B3	W5MQ37	UniProt
LEPOC_B6	W5MNT4	UniProt
LOTGI_A4	V4AD33	UniProt
MNELE_X	ML02521a	NHGRI
MONDO_A1	F7APP4	UniProt
MONDO_A2	F7DZ20	UniProt
MONDO_A3	F6UM70	UniProt
MONDO_A4	F6XVY7	UniProt
MONDO_A5	F7ANR2	UniProt
MONDO_A6	F6QF60	UniProt
MONDO_A7	F7CWR4	UniProt
MONDO_A8	F7EB26	UniProt
MONDO_10	F7E4M9	UniProt
MONDO_B1	F7AG95	UniProt
MONDO_B2	F7EAT1	UniProt
MONDO_B3	XP_007486251	RefSeq
MONDO_B4	F6XJC5	UniProt
MONDO_B6	F7ES42	UniProt
MOUSE_A1	Q60750	UniProt
MOUSE_A2	Q03145	UniProt
MOUSE_A3	P29319	UniProt
MOUSE_A4	Q03137	UniProt
MOUSE_A5	Q60629	UniProt
MOUSE_A6	Q62413	UniProt
MOUSE_A7	Q61772	UniProt
MOUSE_A8	O09127	UniProt
MOUSE_10	Q8BYG9	UniProt
MOUSE_B1	Q8CBF3	UniProt
MOUSE_B2	P54763	UniProt
MOUSE_B3	P54754	UniProt
MOUSE_B4	P54761	UniProt
MOUSE_B6	O08644	UniProt
NEMVE_A4	209449	JGI
NEMVE_A6	128719	JGI
NEMVE_B6	234961	JGI
NEMVE_X	10376	JGI
OIKDI_A7	E4WXV5	UniProt
OIKDI_AX2	E4Y9Q2	UniProt
OIKDI_BX	E4X2Z0	UniProt
OIKDI_AX1	E4XK50	UniProt
OPHHA_A2	V8NVZ2	UniProt
OPHHA_A3	ETE63205+ETE60490+ETE63206	GenBank
OPHHA_A4	ETE64979+ETE64980+ETE60536	GenBank

OPHHA_A5	V8NEI7	UniProt
OPHHA_A6	V8NNM8	UniProt
OPHHA_A7	ETE72989+ETE72990	GenBank
OPHHA_A8	ETE56438+ETE55955	GenBank
OPHHA_B1	ETE61020+ETE64160	GenBank
OPHHA_B2	ETE59899+ETE56285	GenBank
OPHHA_B3	V8NDG3	UniProt
OPHHA_B4	ETE59232+ETE57386+ETE57385	GenBank
ORNAN_A2	F6WYZ6	UniProt
ORNAN_A3	F7EX10	UniProt
ORNAN_A4	F6TGX8	UniProt
ORNAN_A5	F6SA04	UniProt
ORNAN_A6	F6TJ98	UniProt
ORNAN_A7	F7D2V3	UniProt
ORNAN_10	F6ULS0	UniProt
ORNAN_B1	XP_003431449	RefSeq
ORNAN_B3	F7DPU2	UniProt
ORNAN_B4	F7C3F7	UniProt
ORNAN_B6	F7DSW8	UniProt
ORYLA_A2	H2N0S8	UniProt
ORYLA_A3	H2MMA8	UniProt
ORYLA_A4	H2LYR1	UniProt
ORYLA_A5	H2M8D3	UniProt
ORYLA_A6	H2MLG6	UniProt
ORYLA_A7	H2MUM5	UniProt
ORYLA_A8	H2MAH9	UniProt
ORYLA_10	H2M286	UniProt
ORYLA_B1	H2MMP7	UniProt
ORYLA_B2	H2MRL1	UniProt
ORYLA_B3	H2LSJ4	UniProt
ORYLA_B4	H2LRI3	UniProt
ORYLA_B6	H2M8R4	UniProt
OSCCA_A5	g5890.t1	Compagen
OSCCA_A7	g909.t1	Compagen
PELSI_A1	K7FLC7	UniProt
PELSI_A2	K7FBA4	UniProt
PELSI_A3	K7GIS4	UniProt
PELSI_A4	K7FBC9	UniProt
PELSI_A5	K7F4M0	UniProt
PELSI_A6	K7GJM3	UniProt
PELSI_A7	K7F2Z7	UniProt
PELSI_A8	K7GC54	UniProt
PELSI_B1	K7F958	UniProt
PELSI_B2	K7F217	UniProt
PELSI_B3	K7GGF3	UniProt
PELSI_B5	K7G237	UniProt
PETMA_A4	S4RYB1	UniProt
PETMA_A5	S4RX53	UniProt
PETMA_A7	GL477646.1	Ensembl
PETMA_AX1	S4RAK3	UniProt
PETMA_AX2	S4RPX4	UniProt
PETMA_B2	S4RRW4	UniProt
PETMA_BX	S4RIU9	UniProt
SACKO_B2	XP_006821187	RefSeq
SCHMA_B1	G4LYL1	UniProt
SCHMA_BX	G4V6K2	UniProt
STRMM_B2	T1IPM1	UniProt
STRPU_A4	XP_011681770	RefSeq
STRPU_B2	W4ZG41	UniProt
STRPU_B3	XP_011681809	RefSeq
TAEGU_A1	H0ZSP2	UniProt
TAEGU_A2	H0YV01	UniProt
TAEGU_A3	H0ZTE5	UniProt
TAEGU_A4	H0ZBM3	UniProt
TAEGU_A5	H0Z2T4	UniProt
TAEGU_A6	H0ZTF0	UniProt
TAEGU_A7	H0ZQ33	UniProt
TAEGU_A8	H1A3R4	UniProt
TAEGU_10	H0YTG1	UniProt
TAEGU_B1	H0Z935	UniProt
TAEGU_B2	H1A3Q0	UniProt
TAEGU_B3	H0ZJI7	UniProt
TAEGU_B6	H0ZSI9	UniProt
TRIAD_A3	B3RWY9	UniProt
TRIAD_A7	B3RNU8	UniProt
TRICA_B2	A0A139WE15	UniProt
TRISP_A4	E5SP53	UniProt
XENTR_A2	F7C2S3	UniProt
XENTR_A3	F7A3W1	UniProt
XENTR_A4	F6QKP3	UniProt
XENTR_A5	XP_012810832	RefSeq
XENTR_A6	F6UVF6	UniProt
XENTR_A7	F6RSL5	UniProt
XENTR_A8	L7N3P2	UniProt
XENTR_10	A4IGW2	UniProt
XENTR_B1	F6ZV43	UniProt
XENTR_B3	F7CRM0	UniProt
XENTR_B4	F6YTU9	UniProt
XENTR_B5	XP_017946007	RefSeq

ST3 – Ephrin Sequence identifiers

Tree seq ID	DB Seq ID	DB
ALLMI_A1	XP_019352776	RefSeq
ALLMI_A2	A0A151PFZ7	UniProt
ALLMI_A3	XP_006264418	RefSeq
ALLMI_A4	A0A151M8R4	UniProt
ALLMI_A5	XP_006264195	RefSeq
ALLMI_B1	A0A151NNG7	UniProt
ALLMI_B2	A0A151NWW5	UniProt
AMPQE_X	I1G910	UniProt
ANOCA_A1	XP_008123674	RefSeq
ANOCA_A2	H9GF41	UniProt
ANOCA_A5	H9GBX4	UniProt
ANOCA_B1	XP_008123044	RefSeq
ANOCA_B2	G1KAZ8	UniProt
ANOCA_B3	H9GFU6	UniProt
APIME_X	A0A087ZWX7	UniProt
BRAFL_1	128499	JGI
BRAFL_2	208614	JGI
CAEEL_A4	O44516	UniProt
CAEEL_B2	Q9U3M2	UniProt
CAEEL_BX1	Q19475	UniProt
CAEEL_BX2	G5EEE7	UniProt
CALMI_A3	V9LJ33	UniProt
CALMI_A4	V9LKU6	UniProt
CALMI_A5	V9KGD1	UniProt
CALMI_B1	V9L394	UniProt
CALMI_B2	V9L9I2	UniProt
CALMI_B3	V9LD60	UniProt
CAPTE_B3	R7V734	UniProt
CHEMY_A1	M7AU42	UniProt
CHEMY_A2	M7AIH2	UniProt
CHEMY_A3	M7B7U8	UniProt
CHEMY_A5	XP_007059633	RefSeq
CHEMY_B2	M7CF48	UniProt
CHICK_A2	P52802	UniProt
CHICK_A5	P52804	UniProt
CHICK_B1	O73612	UniProt
CHICK_B2	Q9PUJ4	UniProt
CIOIN_A1	F6U2Y7	UniProt
CIOIN_A2	Q4H3M1	UniProt
CIOIN_A3	F6U302	UniProt
CIOIN_A4	F6WPE8	UniProt
CIOIN_B2	Q4H3L8	UniProt
CRAGI_B2	XP_011414826	RefSeq
DANRE_A1A	Q6PGZ9	UniProt
DANRE_A1B	O93431	UniProt
DANRE_A2A	E7FB27	UniProt
DANRE_A2B	P79727	UniProt
DANRE_A3A	F1R755	UniProt
DANRE_A3B	Q90YC5	UniProt
DANRE_A4	A0A0R4IWT4	UniProt
DANRE_A5A	F1QFJ9	UniProt
DANRE_A5B	P79728	UniProt
DANRE_B1	Q90Z33	UniProt
DANRE_B2A	O73874	UniProt
DANRE_B2B	Q90Z32	UniProt
DANRE_B3A	E7FG26	UniProt
DANRE_B3B	Q502Q3	UniProt
DAPPU_A5	E9H642	UniProt
DROME_B	Q9V4E1	UniProt
HELRO_A1	T1F1A9	UniProt
HUMAN_A1	P20827	UniProt
HUMAN_A2	O43921	UniProt
HUMAN_A3	P52797	UniProt
HUMAN_A4	P52798	UniProt
HUMAN_A5	P52803	UniProt
HUMAN_B1	P98172	UniProt
HUMAN_B2	P52799	UniProt
HUMAN_B3	Q15768	UniProt
HYDVU_B1	R9WY58	UniProt
HYDVU_B2	R9WWC9	UniProt
HYDVU_B3	R9X0X4	UniProt
HYMMI_X	A0A068X8Y5	UniProt
IXOSC_A4	B7PNC7	UniProt
LATCH_A1	H3APL0	UniProt
LATCH_A2	XP_005999930	RefSeq
LATCH_A3	H3A5X1	UniProt
LATCH_A4	H3AXQ8	UniProt
LATCH_A5	H3BA27	UniProt
LATCH_B1	H3A9V6	UniProt
LATCH_B2	H3ARJ7	UniProt
LEPOC_A2	W5M0M3	UniProt
LEPOC_A3	W5MIQ6	UniProt
LEPOC_A5	W5MS01	UniProt
LEPOC_B1	W5NBU6	UniProt
LEPOC_B2	W5MZ01	UniProt
LEPOC_B3	W5N7F0	UniProt
LOTGI_A4	V4AIY0	UniProt
MENLE_X	ML03442a	NHGRI
MONDO_A1	F6UJM9	UniProt
MONDO_A2	F7GGW2	UniProt
MONDO_A3	F7B5C7	UniProt
MONDO_A4	F6UJJ3	UniProt
MONDO_A5	F6XSQ4	UniProt
MONDO_B1	F7FJ47	UniProt
MONDO_B2	F7F3Y7	UniProt

MONDO_B3	F6ZNA9	UniProt
MOUSE_A1	P52793	UniProt
MOUSE_A2	P52801	UniProt
MOUSE_A3	O08545	UniProt
MOUSE_A4	O08542	UniProt
MOUSE_A5	O08543	UniProt
MOUSE_B1	P52795	UniProt
MOUSE_B2	P52800	UniProt
MOUSE_B3	O35393	UniProt
NEMVE_1	A7RL54	UniProt
NEMVE_2	A7RU50	UniProt
NEMVE_X	A7SIY5	UniProt
OIKDI_B2	E4XRS2	UniProt
OIKDI_B3	E4XH94	UniProt
OIKDI_BX1	E4X0B4	UniProt
OIKDI_BX2	E4WUF1	UniProt
OIKDI_BX3	E4XGR1	UniProt
OPHHA_A1	V8N767	UniProt
OPHHA_A2	V8NL63	UniProt
OPHHA_A3	V8NHC5	UniProt
OPHHA_A5	V8N8R0	UniProt
OPHHA_B1	V8NGW9	UniProt
OPHHA_B2	V8PA86	UniProt
OPHHA_B3	V8NGT8	UniProt
ORNAN_A1	F6RY19	UniProt
ORNAN_A2	F7BCM7	UniProt
ORNAN_A4	F7FAI9	UniProt
ORNAN_A5	F6SAR9	UniProt
ORNAN_B2	F7GEW6	UniProt
ORYLA_A1	H2M1M9	UniProt
ORYLA_A2	H2MFY2	UniProt
ORYLA_A3	H2M1K4	UniProt
ORYLA_A4	H2M1H8	UniProt
ORYLA_A5	H2LG60	UniProt
ORYLA_B1	H2LW22	UniProt
ORYLA_B2	H2LK03	UniProt
ORYLA_B3	H2LF56	UniProt
PELSI_A2	K7F4C9	UniProt
PELSI_A5	K7FUW9	UniProt
PELSI_B1	K7GIX5	UniProt
PELSI_B2	K7G8I8	UniProt
PELSI_B3	K7G7E9	UniProt
PETMA_B2	S4RR11	UniProt
PETMA_B3	S4RS82	UniProt
SACKO_B1	NP_001164690	RefSeq
SACKO_B2	XP_002737429	RefSeq
SCHMA_X	G4V9G6	UniProt
STRMM_B1	T1JC50	UniProt
STRPU_B1	W4Z6J3	UniProt
TAEGU_A1	H1A0X0	UniProt
TAEGU_A2	H0YRI8	UniProt
TAEGU_A3	H0ZYD6	UniProt
TAEGU_A5	H0YRS1	UniProt
TAEGU_B1	H0YWV3	UniProt
TAEGU_B2	H0ZKL4	UniProt
TRICA_B	D6W6N4	UniProt
TRICA_B2	D6WRM8	UniProt
TRISP_B2	E5S2A6	UniProt
TRISP_BX1	E5S1E0	UniProt
TRISP_BX2	E5S299	UniProt
TRISP_BX3	E5S2A4	UniProt
XENTR_A1	F7BTE2	UniProt
XENTR_A2	B1WAY1	UniProt
XENTR_A3	F6QJL2	UniProt
XENTR_A4	XP_004920759	RefSeq
XENTR_A5	Q28H39	UniProt
XENTR_B1	F7B9D9	UniProt
XENTR_B2	F6Z6U9	UniProt
XENTR_B3	A8KBG7	UniProt
